# Bats (Chiroptera) from Madidi National Park: an integrative assessment from field surveys, museum collections, and historical records

**DOI:** 10.3897/zookeys.1296.206387

**Published:** 2026-11-05

**Authors:** Lizette Siles, Marisol Hidalgo-Cossio, Nuria Bernal-Hoverud, Mauricio Ocampo, Robert B. Wallace

**Affiliations:** 1 Área de Mastozoología, Museo de Historia Natural Alcide d’Orbigny, Calle Potosí 1458, Cochabamba, Bolivia Área de Mastozoología, Museo de Historia Natural Alcide d’Orbigny Cochabamba Bolivia; 2 Colección Boliviana de Fauna, Museo Nacional de Historia Natural, Cota Cota Calle 27, La Paz, Bolivia Colección Boliviana de Fauna, Museo Nacional de Historia Natural La Paz Bolivia; 3 Wildlife Conservation Society, Bolivia Program, Calle 22 de Calacoto 8232, La Paz, Bolivia Wildlife Conservation Society, Bolivia Program La Paz Bolivia; 4 Department of Biological Sciences, Texas Tech University, Lubbock, Texas 79409, USA Department of Biological Sciences, Texas Tech University Lubbock United States of America; 5 Red de Investigadores en Herpetología – Bolivia, La Paz, Bolivia Red de Investigadores en Herpetología – Bolivia La Paz Bolivia; 6 Unidad de Zoología, Instituto de Ecología, Universidad Mayor de San Andrés, Casilla 10077, Correo Central, La Paz, Bolivia Unidad de Zoología, Instituto de Ecología, Universidad Mayor de San Andrés La Paz Bolivia

**Keywords:** Acoustic sampling, Bolivia, ecoregions, museum collections, Neotropics, species richness, species turnover

## Abstract

The Neotropical region harbors the greatest biodiversity on Earth, and bats are often the most species-rich mammalian group in local communities. Bolivia’s Madidi National Park is considered one of the most biodiverse protected areas in the world, yet knowledge of its bat fauna has historically remained limited and fragmented. The most comprehensive assessment of bat diversity in Madidi to date is presented, combining historical baseline data from museum collections and literature with field surveys conducted between 2015 and 2017. Fieldwork across 12 localities covered an elevational range from 198 to 4,814 m and combined mist-netting, harp-trapping, roost searches, and acoustic sampling. We confirmed the occurrence of 100 bat taxa, including 99 recognized species and one potentially undescribed species of *Histiotus*, added 25 new records for Madidi, and seven new country records for Bolivia. The Sub–Andean and Pre–Andean Amazon Forests are the most diverse ecoregions and shared the most similar bat assemblages. Overall, we observed high species turnover among ecoregions. With 99 confirmed species, Madidi currently harbors one of the highest documented bat richness among protected areas worldwide, representing 68% of Bolivia’s bat fauna. This reinforces Madidi’s status as a global conservation priority and provide a verified baseline for future bat research.

## Introduction

The Neotropics is home to the greatest biodiversity on Earth (Antonelli et al. 2018). Although multiple factors have contributed to this exceptional richness, the uplift of the Andes is considered one of the main drivers, acting as a species pump by creating new habitats and promoting biotic isolation and diversification (Hoorn et al. 2022). Consequently, biodiversity surveys are especially important in these regions, where scientific knowledge often remains limited and fragmented (Amano and Sutherland 2013; Hughes et al. 2021). In Bolivia, research efforts frequently focus on large, charismatic fauna or wildlife management, leaving a significant knowledge gap for smaller, more diverse vertebrate groups such as bats (Tarifa and Aguirre 2009), which are often the most diverse mammal group in tropical regions (Hughes et al. 2021).

**Figure 1.**
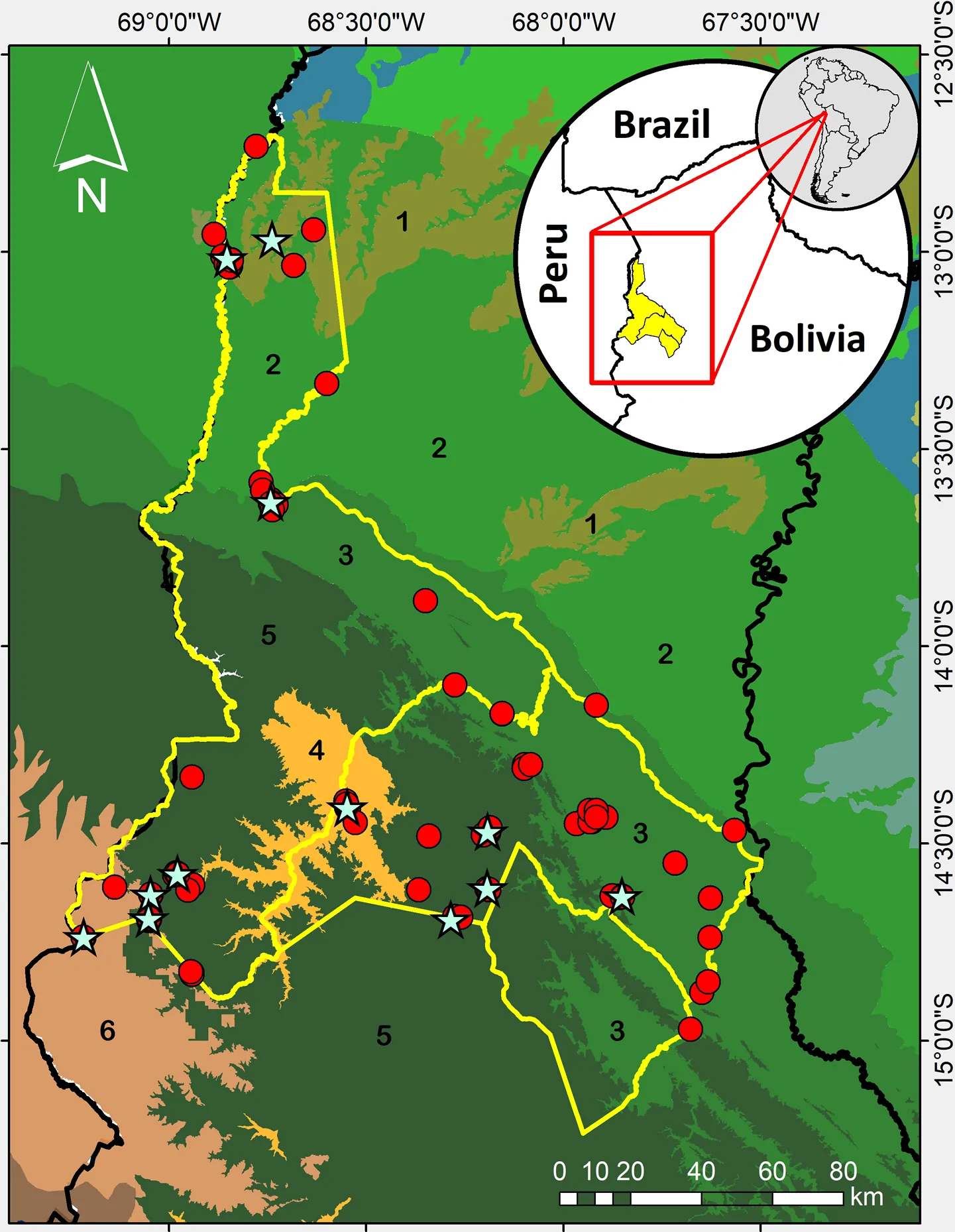
Map of the study area. Madidi National Park and Natural Area of Integrated Management (yellow outline), Identidad Madidi fieldwork sites (light blue stars), bat records from the baseline and field surveys (red circles), and ecoregions (numbered): Cerrado Paceño (1), Pre–Andean Amazon Forests (2), Sub–Andean Amazon Forests (3), Inter–Andean Dry Forests (4), Yungas (5), and High Andean Vegetation (6).

Madidi National Park and Natural Area of Integrated Management (hereafter Madidi) is recognized as one of the most biodiverse protected areas in the world (Salinas and Wallace 2012; Ocampo et al. 2024; Aparicio et al. 2025). Spanning an elevational gradient from 180 to 6,044 m, it protects a remarkable representation of ecosystems, including six distinct ecoregions following the official national classification framework of Ibisch et al. (2003): Cerrado Paceño, Pre–Andean Amazon Forests, Sub–Andean Amazon Forests, Inter–Andean Dry Forests, Yungas, and High Andean Vegetation (Fig. 1). These ecoregions represent broad ecological units containing diverse vegetation types. Madidi is estimated to harbor 3.5% of the world’s known vertebrate diversity, positioning it among the world’s highest conservation priorities (Identidad Madidi and SERNAP 2017, 2019, 2020; Miranda-Chumacero et al. 2022; Ocampo et al. 2024; Aparicio et al. 2025). Despite its biological importance, prior knowledge of the park’s fauna was scattered and limited, or restricted to a few accessible localities, providing only an incomplete understanding of its true diversity.

Bats represent a major component of this richness, with Neotropical lowland forests potentially supporting up to 100 sympatric species through niche partitioning (Voss and Emmons 1996). Obtaining an accurate inventory of this group requires strong taxonomic expertise, the collection of voucher specimens that serve as an essential resource for future morphological and molecular revisions, and the integration of acoustic tools, especially for species that are difficult to detect using traditional methods (Appel et al. 2021).

To address these knowledge gaps, the Identidad Madidi initiative (https://madidiid.org) was conducted as a multi-institutional scientific expedition from 2015 to 2017 to document the park’s biodiversity across its elevational gradient and promote environmental awareness. Here, we present the most comprehensive assessment of bat diversity in Madidi to date, combining results from multi-method field surveys across 12 study sites with a thorough compilation of historical records. By synthesizing these data, we aim to confirm Madidi’s global significance for mammalian conservation and provide a verified baseline for future research.

## Materials and methods

### Study area

Madidi is located in the Department of La Paz, in northwestern Bolivia, within the upper Amazon River basin (Fig. 1). Designated as a national park in 1995, it is the third-largest protected area in Bolivia, covering 18,957.4 km^2^ and ranging in elevation from 180 to 6,044 m. Following the national classification of Ibisch et al. (2003), Madidi encompasses portions of six ecoregions (Fig. 1). The elevational ranges and predominant vegetation of these ecoregions, as described by Ibisch et al. (2003), are as follows: 1) Cerrado Paceño (180–500 m), with oligotrophic savannas dominated by tough grasses, sedges, and shrubs, locally including mounded areas, forest islands, and sedge and palm swamps; 2) Pre–Andean Amazon Forests (150–500 m), with tall (30–45 m), predominantly evergreen humid forests, including emergent trees and trees with buttress roots; 3) Sub–Andean Amazon Forests (500–1,000 m), with tall (30–35 m), multi-layered humid evergreen forests rich in epiphytes and lianas; 4) Inter–Andean Dry Forests (500–3,300 m), with deciduous dry forests (10–20 m) comprising diverse forest formations, including woodlands with columnar cacti; 5) Yungas (1,000–4,200 m), with medium low-stature (5–30 m) humid evergreen forests, including cloud forests (Ceja del Monte) and, at higher elevations, evergreen shrublands and anthropogenic grasslands of the Páramo Yungueño; and 6) High Andean Vegetation (4,000–>5,000 m), with open grasslands on rocky terrain, low grasslands with or without cushion plants, and vegetation growing in rock crevices. Owing largely to its pronounced elevational gradient and associated habitat diversity, Madidi is considered one of the most biodiverse protected areas in the region (Salinas and Wallace 2012; Identidad Madidi and SERNAP 2017, 2019, 2020).

### Baseline

We compiled a database of bat records from publications, museum collections, and unpublished literature, including technical reports and research theses (Suppl. material 1). The database was developed and updated between 2015 and 2026 to incorporate new records and taxonomic changes. Local natural history collections included the Colección Boliviana de Fauna (**CBF**, Museo Nacional de Historia Natural and Instituto de Ecología UMSA), and the Museo de Historia Natural Noel Kempff Mercado (**MNKM**), whereas international collections included the American Museum of Natural History (**AMNH**), the Museum of Southwestern Biology (**MSB**), the University of Michigan Museum of Zoology (**UMMZ**), and the Smithsonian Institution, National Museum of Natural History (**USNM**). To standardize the dataset and determine which records could be considered confirmed occurrences in Madidi, we applied the following criteria: 1) scientific names were updated according to the most recent version of batnames.org (Simmons and Cirranello 2026) and relevant recent primary taxonomic revisions; 2) when taxonomic revisions restricted a species’ distribution and excluded Bolivia, records were reassigned to the corresponding valid Bolivian taxon; 3) for taxonomic splits involving the elevation of subspecies to species rank, records were assigned to the newly recognized species only when the original source explicitly identified the subspecies. Records lacking sufficient taxonomic detail to determine the species were excluded; 4) we examined voucher specimens of *Sturnira lilium* and *S. giannae* in both national collections, as well as *Anoura* specimens deposited at CBF; 5) for other taxa, identifications reported in collection databases were accepted without independent examination of the specimens. For published and unpublished records associated with deposited specimens, we prioritized the current identification in the collection database because it reflects more recent curatorial updates; 6) a research thesis containing sonograms was evaluated against currently available diagnostic call characteristics, and incorrect acoustic identifications were excluded; and 7) to account for georeferencing inaccuracies in historical records and the natural dynamics of river meanders that define portions of the park’s boundaries, we applied a 1-km buffer around the protected area when assessing spatial eligibility. A record was considered a confirmed occurrence in Madidi if it met the taxonomic and spatial criteria.

### Fieldwork

During the Identidad Madidi expedition (https://madidiid.org/), we sampled bats from June 2015 to November 2017 at 12 localities representing different elevations, ecoregions and habitats (Table 1). In addition, one specimen was collected opportunistically at an additional locality, Puina Bajo (14.6107°S, 69.1372°W, 3,837 m a.s.l.), where formal bat sampling was not conducted, although the site was visited during the expedition.

**Table 1.** Study sites surveyed during the Identidad Madidi expedition, including geographic coordinates (decimal degrees), elevation, ecoregion, and sampling effort for mist-netting and acoustic sampling. At Alto Madidi, mist-net effort was 14,096.3 m^2^xh in Pre-Andean Amazon Forests and 90 m^2^xh in Sub-Andean Amazon Forests. All acoustic sampling effort at this locality was conducted in Pre-Andean Amazon Forests.

**Study sites**	**Latitude, Longitude**	**Elevation (m a.s.l.)**	**Mist-net effort (m^2^xh)**	**Acoustic effort (h)**	**Ecoregions**
Heath Pampa	12.9683°S, 68.7383°W	198	3,198.8	18.4	Cerrado Paceño
Heath Bosque	13.0163°S, 68.852°W	198	9,770.6	17.6	Pre–Andean Amazon Forests
Alto Madidi	13.6332°S, 68.7417°W	252	14,186.3	14.1	Sub, Pre–Andean Amazon Forests
Hondo Alto	14.6335°S, 67.8509°W	301	13,494	11.3	Sub–Andean Amazon Forests
Sipia	14.4081°S, 68.5475°W	740	10,814.9	0.5	Inter–Andean Dry Forests
Sarayoj	14.6134°S, 68.1924°W	1,158	3,501.8	4.8	Yungas
Mamacona	14.4697°S, 68.1921°W	1,566	6,173.25	12.1	Yungas
Machariapo	14.6921°S, 68.2853°W	1,763	10,767	3.5	Yungas
Cargadero	14.5772°S, 68.9778°W	2,155	7,453	10.5	Yungas
Chullo	14.6896°S, 69.0514°W	2,851	2,663.4	21	Yungas
Isañujoy	14.6288°S, 69.0464°W	3,460	990	13.6	Yungas
Chokollo	14.7369°S, 69.2156°W	4,814	1,783.1	-	High Andean Vegetation

**Figure 2.**
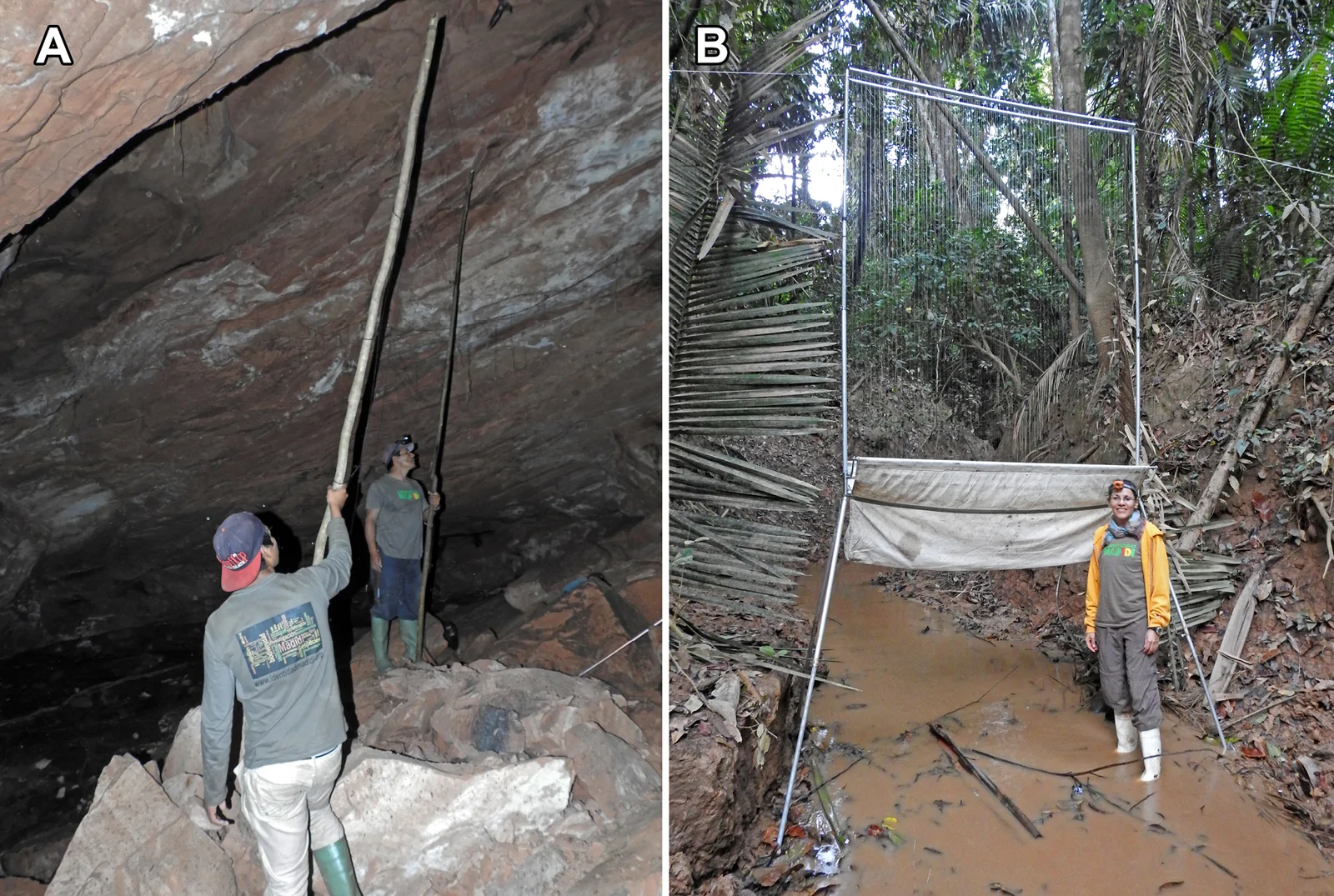
Complementary survey methods during the Identidad Madidi expedition. **A**. Cave survey; **B**. Harp-trap deployment.

To capture bats, we deployed understory mist nets (Avinet, U.S.A., and Ecotone, Poland) of 6, 9, and 12 m along trails, streams, lakes, near fruiting or flowering plants, or in other locations where bat activity was expected to be high. Occasionally, we placed mist nets higher in open areas, with the bottom edge of the net positioned 2 m above the ground. The total mist-netting effort across all sampled localities, calculated as net surface area multiplied by the number of hours open, was 84,796.15 m^2^xh (Straube and Bianconi 2022). Effort varied among sites (Table 1) and ecoregions, being highest in Yungas (31,548.5 m^2^xh), followed by the Pre-Andean Amazon Forests (23,866.9 m^2^xh), Sub-Andean Amazon Forests (13,584 m^2^xh), Inter–Andean Dry Forests (10,814.9 m^2^xh), Cerrado Paceño (3,198.8 m^2^xh), and High Andean Vegetation (1,783.1 m^2^xh). To complement mist netting, we conducted opportunistic roost searches at all sites, focusing primarily on accessible structural roosts (e.g., caves, hollow trees, and buildings), with support from local guides and project members. Additionally, we used a harp-trap at three sites (3 nights at Heath Bosque, 7 nights at Chullo, and 6 nights at Chokollo) (Fig. 2).

Up to five specimens per species or morphotype were collected in accordance with the guidelines by Sikes et al. (2011) under government research permit MMAYA/VMABCCGDF/DGBAP/UVSAP No. 354/2014. Most specimens were preserved in alcohol, with skulls extracted and cleaned. A few were dry-preserved as skins and skeletons. We deposited all specimens in the Colección Boliviana de Fauna (CBF) in La Paz, Bolivia (specimen vouchers are listed in Suppl. material 1).

Preliminary identifications of captured bats were made in the field and later confirmed in the laboratory after the skulls were cleaned. Taxonomic identifications were based on the criteria published by Gardner (2007) and Díaz et al. (2021). In the field, we recorded body measurements, weight, age, and reproductive condition. Body measurements (except forearm length) were taken with a metal ruler, and forearm length and skull measurements were taken with a digital caliper (Mitutoyo, Japan) to the nearest 0.1 mm, following Lira et al. (1994). Weight was measured with a spring scale (Pesola, Switzerland). Age was estimated by transilluminating the wing and examining the closure rate of the cartilaginous epiphyseal growth plate in the long phalanges of the fifth finger (Brunet-Rossinni and Wilkinson 2009). Skull measurements were taken in the laboratory. The reproductive condition of adult female bats was recorded and categorized as pregnant, lactating, post-lactating, or non-reproductive. These observations are provided as natural history data, with counts by species in Suppl. material 2.

We recorded echolocation calls using the Anabat Walkabout ultrasonic detector (Titley Scientific, Australia) at all sites except Chokollo, where it malfunctioned. The detector was set to automatic mode and left to record in open areas, such as rivers, lagoons, trails, and cultivated sites, often simultaneously with mist-netting efforts. Occasionally, we recorded bat calls manually when bats could be observed and time allowed. Total acoustic sampling effort was 127.4 hours, distributed among ecoregions as follows: Yungas (65.5 h), Pre-Andean Amazon Forests (31.7 h), Cerrado Paceño (18.4 h), Sub-Andean Amazon Forests (11.3 h), and Inter-Andean Dry Forests (0.5 h). Although acoustic sampling was attempted in High Andean Vegetation, the resulting files were corrupted and could not be analyzed. Sampling effort by study site is provided in Table 1.

### Acoustic analyses

Recordings were saved to memory cards in two formats, full-spectrum WAV files and zero-crossing (ZC) files, which are specific to the Anabat system. In a few cases (e.g., Chullo), only ZC files were available. ZC files were analyzed using AnalookW (v. 4.2g 2016, Chris Corben), whereas full-spectrum WAV files were examined in Anabat Insight (Titley Scientific, v. 2.0.8-0-g4157d1f), which was also used to generate the figures presented in the Results. In Anabat Insight, we examined the full-spectrum recordings using spectrograms, harmonics, and oscillograms. In some cases, full-spectrum WAV files contained valuable information that was not present in their corresponding ZC files. When necessary, these WAV files were converted to ZC format using AnabatConverter (https://bertrik.sikken.nl/anabat/) to enable parameter measurement. Acoustic parameters were measured using AnalookW, which allowed us to filter noise and isolate calls or pulses. Species identification was based on comparisons of call structure, harmonics, and parameter values from search-phase calls, using published references including Arias-Aguilar et al. (2018), Miller et al. (2023), and Rodríguez-San Pedro et al. (2023). For a few species, identifications were additionally supported by comparisons with reference recordings from locally identified specimens, particularly for taxa with similar acoustic characteristics. Because acoustic parameters may vary within species, identifications were based on more than a single frequency measurement. The following abbreviations were used to describe calls: Modulated Frequency (FM), Constant Frequency (CF), *quasi*-Constant Frequency (qCF), Frequency of Maximum Energy of the call (FME), Maximum Frequency of the call (Fmax), Minimum Frequency of the call (Fmin), Call Duration (Dur). Subscript u and d indicate an upward or downward frequency, and subscripts 1, 2, and 3 indicate call types I, II, and III, respectively (Arias-Aguilar et al. 2018). In AnalookW, Fmax, Fmin, and Dur were measured using the Measurements tool. Because AnalookW does not provide a direct measurement of FME, we measured the frequency corresponding to the maximum value in the Cycles Graph (F_CG) and used it as an operational proxy for the FME reported in published acoustic descriptions. F_CG is derived from zero-crossing analysis and is not equivalent to FME obtained from a full-spectrum analysis. For *Diclidurus*, we also measured the characteristic frequency of the dominant harmonic (Fc), defined as the frequency at the right-hand end of the portion of the call with the lowest absolute slope, and the characteristic slope (Sc), measured in octaves per second (Ops) (Miller et al. 2023). All frequencies were measured in kilohertz (kHz) and call duration in milliseconds (ms). Acoustic voucher recordings documenting the new distributional records were deposited in Zenodo (doi: 10.5281/zenodo.21300753), and a description of the dataset is provided in Suppl. material 3.

### Data analyses

Using the fieldwork data, we generated record-based and sample-based rarefaction curves, calculated nonparametric species richness estimators, and quantified dissimilarity among ecoregions. To combine capture and acoustic data, we defined analytical records as follows: each captured individual counted as one record, whereas species detected acoustically on a given night counted as one species-night record, regardless of the number of passes, to avoid overcounting repeated detections. Thus, acoustic detections were included in the analyses as species-night records rather than as captured individuals. Analyses were conducted in RStudio (R Core Team 2022), using the vegan and BiodiversityR packages. Record-based rarefaction curves were generated with the rarefy function, sample-based rarefaction curves with specaccum, the abundance-based richness estimator ACE (Abundance-based Coverage Estimator) values with estimateR, and the incidence-based species richness estimators (Chao2 and Jackknife2) with specpool.

To evaluate compositional differences among ecoregions, we calculated Whittaker’s beta diversity index using the betadiver function from the BiodiversityR package. The index is calculated as:

βW=S/α−1

where S is the total number of species and α is the average number of species per ecoregion. We visualized dissimilarities among ecoregions using a dendrogram and a ternary diagram. Because Whittaker’s index can increase with sample size as S increases while α remains relatively constant, the ternary diagram was used to facilitate pairwise comparisons among ecoregions.

Sampling effort varied because field sites were primarily selected to explore previously unsampled areas rather than to ensure balanced sampling across ecoregions. Given that imperfect detectability and species undersampling can influence observed richness and compositional turnover in tropical bat assemblages (Meyer et al. 2011, 2014), ecoregional comparisons of species richness and beta diversity should be interpreted as baseline characterizations rather than complete assessments of community composition.

## Results

### Species richness and composition

In total, 100 bat taxa were confirmed to occur in Madidi, including 99 recognized species and one potentially undescribed species of *Histiotus* (Table 2). From the baseline, we compiled a database containing 364 records representing 75 species confirmed within the protected area. During fieldwork, we captured 820 individuals representing 69 species and acoustically recorded 28 species, 14 of which were detected exclusively through acoustic sampling. This resulted in a total of 83 species recorded during the three-year expedition. Most taxa were documented in both the baseline and fieldwork datasets (58 species), whereas 17 species were exclusive to the baseline, and 25 were recorded only during fieldwork, representing new records for Madidi. We identified seven new country records, including one previously overlooked museum specimen from the baseline dataset deposited at USNM, two documented through collected vouchers during fieldwork, and four detected acoustically. In addition, a specimen of *Gardnerycteris koepckeae* was recorded during fieldwork; however, this species is not included among the new country records because its occurrence had been reported previously (Siles and Wallace 2021). We also recorded reproductive condition of adult females captured during fieldwork, providing natural history observations for 38 species (Suppl. material 2).

**Table 2.** Bat taxa recorded in Madidi. The potentially undescribed species (†) is listed as sp. Rec = type of record (fw = Identidad Madidi fieldwork, bl = baseline data, bo = both); IUCN = conservation status according to IUCN Red List (2026) (LC = Least Concern, NT = Near Threatened, DD = Data Deficient, NE = Not Evaluated); BCS = Bolivian conservation status (VU = Vulnerable, NT = Near Threatened, DD = Data Deficient, NE = Not Evaluated) (Acosta et al. 2026); ecoregions (CP = Cerrado Paceño, PAAF = Pre–Andean Amazon Forests, SAAF = Sub–Andean Amazon Forests, IADF = Inter–Andean Dry Forests, YU = Yungas, HAV = High Andean Vegetation); records within ecoregions (a = acoustic detection, ca = captured and released, co = collected voucher); * = new record for the park, ** = new record for Bolivia.

**Species**	**Rec**	**IUCN**	**BCS**	**CP**	**PAAF**	**SAAF**	**IADF**	**YU**	**HAV**
Emballonuridae									
*Cormura brevirostris* **	bo	LC	NE		a	a			
*Diclidurus isabella* **	fw	LC	NE	a	a				
*Peropteryx kappleri* **	bo	LC	NE	a	a	a			
*Peropteryx macrotis* *	fw	LC	NE	a	a	a		a, co	
*Rhynchonycteris naso*	bo	LC	NE		a, co	a, co		a	
*Saccopteryx bilineata*	bo	LC	NE		a	a, co			
*Saccopteryx canescens*	bo	LC	DD		a	a			
*Saccopteryx leptura* *	fw	LC	NE		a				
Molossidae									
*Cynomops planirostris*	bo	LC	NE			a, co		a	
*Eumops auripendulus*	bo	LC	NE			co	co		
*Eumops patagonicus*	bl	LC	NE			co			
*Eumops perotis* *	fw	LC	DD	a	a	a		a	
*Molossus coibensis* **	bl	LC	NE			co			
*Molossus currentium* **	fw	LC	NE	a	a			a	
*Molossus molossus*	bo	LC	NE	a	a, co	a, ca, co	a, ca, co	a	
*Molossus rufus*	bo	LC	NE	a	a	a, co			
*Nyctinomops laticaudatus*	bo	LC	NE		a	a, co		a	
*Promops centralis*	bo	LC	DD	a, co	a, co	a		a	
*Promops nasutus* *	fw	LC	NE			a		a	
*Tadarida brasiliensis* *	fw	LC	NE		a	a		a	
Noctilionidae									
*Noctilio albiventris*	bo	LC	NE		co	a, co			
*Noctilio leporinus*	bo	LC	NE		a, ca, co	a, ca, co			
Phyllostomidae									
*Anoura aequatoris* **	fw	LC	NE				co		
*Anoura caudifer*	bo	LC	NE			co		co	
*Anoura fistulata* **	fw	DD	NE					co	
*Anoura geoffroyi* *	fw	LC	NE					co	
*Anoura peruana* *	fw	LC	NE					co	
*Artibeus lituratus*	bo	LC	NE	ca	ca, co	a, ca, co			
*Artibeus obscurus*	bo	LC	NE		ca, co	a, ca, co			
*Artibeus planirostris*	bo	LC	NE	ca	ca, co	ca, co	ca, co	ca, co	
*Carollia benkeithi*	bo	LC	NE		ca, co	ca, co		co	
*Carollia brevicauda*	bo	LC	NE	ca	ca, co	a, ca, co	co	ca, co	
*Carollia manu*	bo	LC	DD			ca, co		ca, co	
*Carollia perspicillata*	bo	LC	NE	ca	ca, co	ca, co	ca, co	co	
*Chiroderma salvini*	bo	LC	NE		co	co		ca, co	
*Chiroderma trinitatum*	bo	LC	NE		co	ca, co			
*Chiroderma villosum*	bo	LC	NE			ca, co			
*Choeroniscus minor*	bl	LC	DD			ca, co			
*Chrotopterus auritus*	bo	LC	NE		ca, co	ca, co	ca		
*Dermanura anderseni*	bo	LC	NE		co	co			
*Dermanura glauca*	bo	LC	NE	co	co	co	co	co	
*Dermanura gnomus* *	fw	LC	DD		co				
*Desmodus rotundus*	bo	LC	NE		ca, co	ca, co	ca, co	ca, co	
*Diphylla ecaudata* *	fw	LC	NT					co	
*Enchisthenes hartii*	bo	LC	NE		co	co		ca, co	
*Gardnerycteris crenulata*	bl	LC	NE		ca	co			
*Gardnerycteris koepckeae*	fw	DD	DD					co	
*Glossophaga soricina*	bo	LC	NE		co	ca, co	co		
*Glyphonycteris daviesi*	bl	LC	VU			co			
*Hsunycteris thomasi*	bl	LC	NE		co	co			
*Lionycteris spurrelli*	bl	LC	DD			co			
*Lophostoma brasiliense*	bo	LC	NE		co	co			
*Lophostoma carrikeri*	bl	LC	NT			ca			
*Lophostoma silvicola*	bo	LC	NE	ca	ca, co	ca, co	ca, co		
*Mesophylla macconnelli*	bo	LC	NE		ca, co	ca, co	co		
*Micronycteris hirsuta*	bl	LC	NE			co			
*Micronycteris megalotis*	bo	LC	NE			ca		co	
*Micronycteris minuta*	bo	LC	NE		co		co		
*Phylloderma stenops*	bl	LC	NE			co			
*Phyllostomus elongatus*	bo	LC	NE	ca	ca, co	a, ca, co	ca, co		
*Phyllostomus hastatus*	bo	LC	NE		ca, co	ca, co			
*Platyrrhinus brachycephalus*	bo	LC	NE		co	co			
*Platyrrhinus incarum*	bo	LC	NE		co	co	ca, co	co	
*Platyrrhinus infuscus*	bo	LC	NE		co	ca, co		co	
*Platyrrhinus lineatus*	bl	LC	NE		co				
*Platyrrhinus masu* *	fw	LC	NE					co	
*Platyrrhinus umbratus* *	fw	DD	DD			co		co	
*Rhinophylla pumilio*	bo	LC	NE		co	ca, co			
*Sturnira erythromos*	bo	LC	NE			ca, co		ca, co	
*Sturnira giannae*	bo	NE	NE		co	co	co	co	
*Sturnira lilium*	bo	LC	NE		co			co	
*Sturnira magna* *	fw	LC	NE				co	ca, co	
*Sturnira oporaphilum*	bo	LC	NE		co	ca, co	ca, co	ca, co	
*Sturnira tildae*	bo	LC	NE			co			
*Tonatia maresi*	bo	NE	NE			ca, co			
*Trachops cirrhosus*	bo	LC	NE		ca, co	ca, co			
*Trinycteris nicefori*	bl	LC	VU		co	ca, co			
*Uroderma bilobatum*	bo	LC	NE		co	ca, co			
*Uroderma magnirostrum*	bo	LC	NE		ca, co	ca, co			
*Vampyressa thyone*	bl	LC	NE			ca, co			
*Vampyriscus bidens*	bo	LC	NE		co	ca, co			
*Vampyrodes caraccioli*	bl	LC	DD			ca, co			
*Vampyrum spectrum* *	fw	NT	VU		ca			ca, co	
Thyropteridae									
*Thyroptera discifera*	bl	LC	DD			ca			
*Thyroptera tricolor*	bo	LC	DD		co	co			
Vespertilionidae									
*Histiotus montanus* *	fw	LC	NE					a	
*Histiotus* sp. †	fw	NE	NE					co	co
*Histiotus velatus*	bl	DD	NE					co	
*Lasiurus blossevillii* *	fw	LC	NE		a	co	co	a	
*Lasiurus ega*	bl	LC	NE		co	co			
*Lasiurus villosissimus* *	fw	NE	NE		a	a		a	co
*Myotis albescens*	bo	LC	NE		a, co	a, co	co	a, co	
*Myotis keaysi*	bo	LC	NE		a	a	co	a, co	
*Myotis levis*	bo	LC	NT		co		co	co	
*Myotis nigricans*	bo	LC	NE	a	a, co	a, co	co	a, co	
*Myotis riparius*	bo	LC	NE		a, co	ca, co	co	co	
*Neoeptesicus andinus* *	fw	LC	NE					a, co	
*Neoeptesicus brasiliensis* *	fw	LC	DD		co				
*Neoeptesicus chiriquinus* *	fw	LC	DD		a			a	
*Neoeptesicus furinalis*	bo	LC	NE	a, co	a	a		a, co	

No Mormoopidae were recorded during our fieldwork. A previous acoustic record attributed to *Pteronotus gymnonotus* from Madidi was reported by Terán (2004) and subsequently included by Siles (2007). We examined the sonogram for this record and found that the call structure is more consistent with *Noctilio* than with Mormoopidae. We therefore do not consider this record sufficient evidence for the occurrence of this family in Madidi.

The Sub–Andean Amazon Forests and Pre–Andean Amazon Forests had the highest observed species richness, with 77 and 66 species, respectively. The Yungas contained 49 species, representing 49.5% of the species recorded in the study. In contrast, the Inter–Andean Dry Forests, Cerrado Paceño, and High Andean Vegetation had comparatively low observed species richness, with 24, 17, and 2 species, respectively.

### Species accumulation, richness estimators, and ecoregion turnover

**Figure 3.**
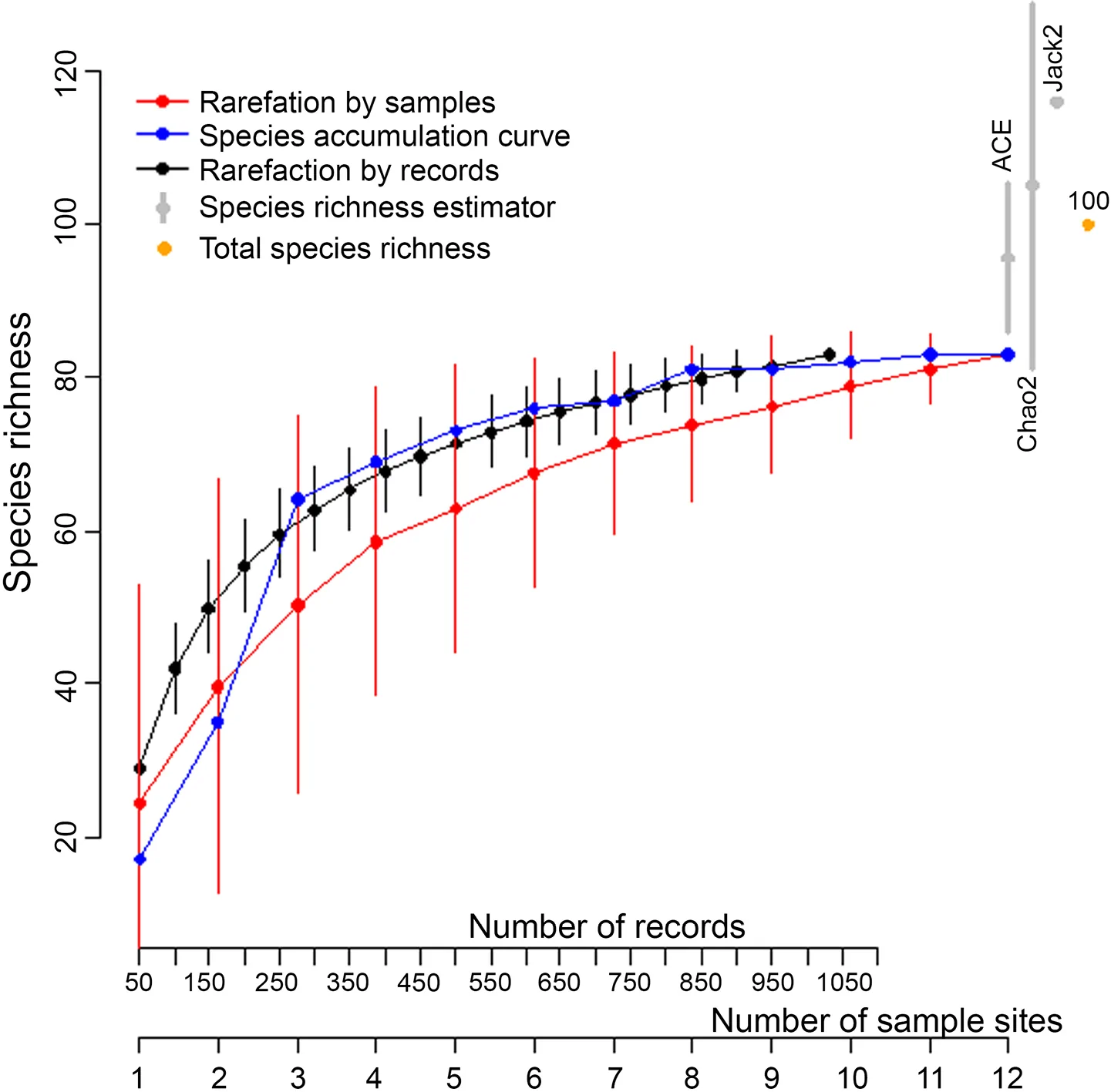
Species accumulation curves based on fieldwork data collected in Madidi. The record-based rarefaction curve represents species accumulation computed for every 50 records, comprising physical captures and acoustic species-night records, whereas the sample-based rarefaction curve represents species accumulation across sampled sites. Sampling effort at each site is provided in Table 1. Total species richness is the combined number of species recorded from the baseline and fieldwork datasets.

The species accumulation curve showed a rapid increase in richness up to the third sampled site, after which the rate of accumulation slowed down considerably toward the final sites, approaching an asymptote (Fig. 3). However, the sample-based rarefaction curve indicated that sampling had not yet fully reached an asymptote. The record-based rarefaction curve was based on 1,032 records, comprising 820 physical captures and 212 acoustic species-night records, which documented 83 species during fieldwork. The non-parametric species richness estimators predicted higher richness values than those observed during fieldwork alone. Among these, the Chao2 estimator predicted 105 ± 11.9 species and was the closest to the combined richness obtained from the baseline and fieldwork datasets. ACE estimated 95.67 ± 4.84 species, whereas Jackknife2 predicted the highest richness value, with 115.91 species.

**Figure 4.**
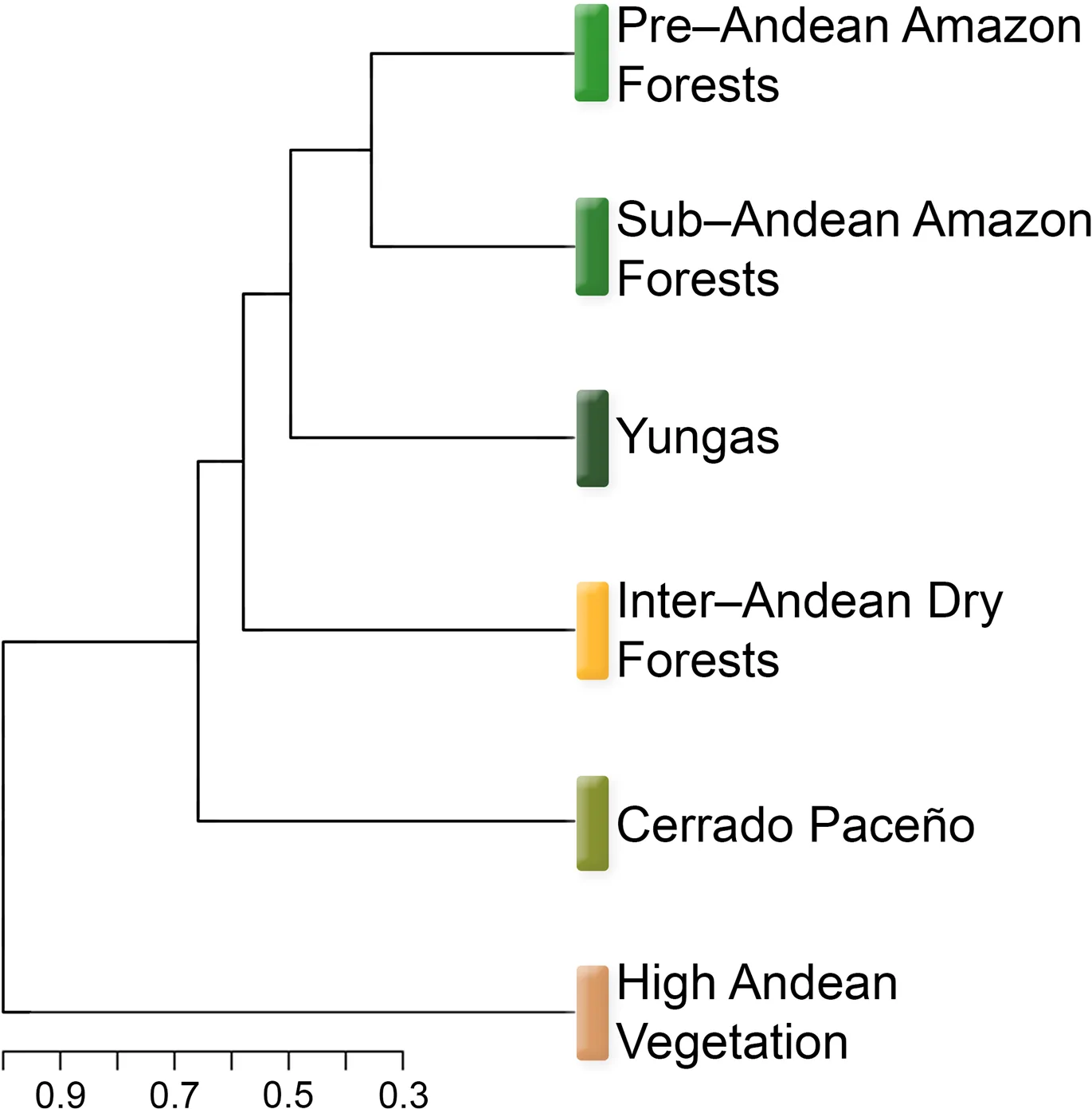
Dendrogram of compositional dissimilarity among ecoregions in Madidi. Colors correspond to those shown in Fig. 1, and the scale represents the degree of compositional difference among ecoregions.

The dendrogram based on the dissimilarity index (values in Suppl. material 4) showed that the Pre–Andean Amazon Forests and the Sub–Andean Amazon Forests were the most similar ecoregions, sharing 55 species, and that both were more similar to the Yungas than to the remaining ecoregions (Fig. 4). In contrast, the High Andean Vegetation was highly distinct from all other ecoregions, despite being geographically closest to Yungas.

**Figure 5.**
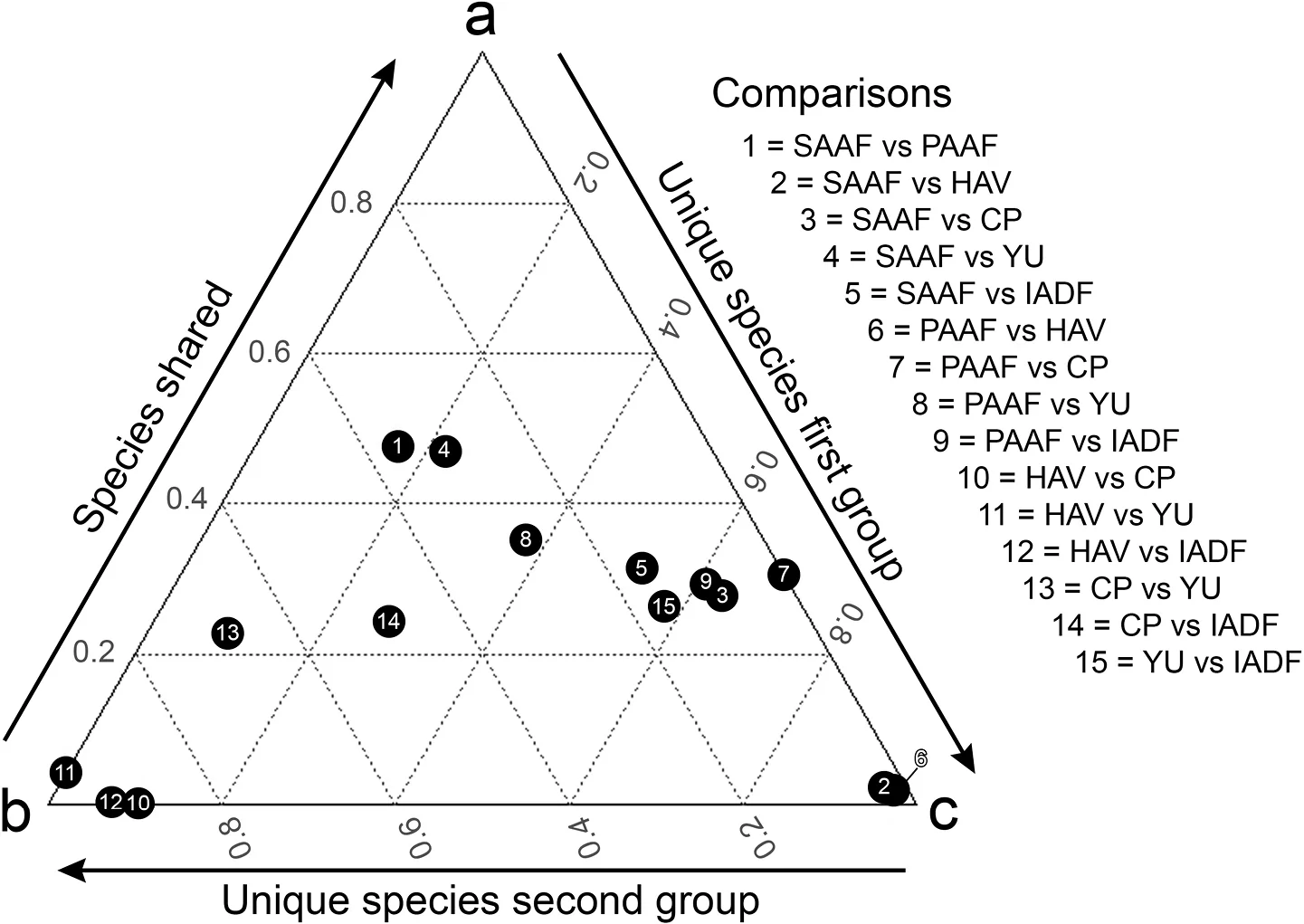
Ternary diagram showing pairwise composition among ecoregions in Madidi. a represents the number of species shared between two ecoregions, and b and c represent the numbers of species unique to each ecoregion. Ecoregions: CP = Cerrado Paceño, PAAF = Pre–Andean Amazon Forests, SAAF = Sub–Andean Amazon Forests, IADF = Inter–Andean Dry Forests, YU = Yungas, HAV = High Andean Vegetation.

The ternary diagram (Fig. 5) further demonstrated that several pairwise comparisons among ecoregions shared very few species. Comparisons 2, 6, 10, 11, and 12, all involving High Andean Vegetation, showed minimal overlap in species composition. Comparisons 1 and 4, corresponding to Sub–Andean Amazon Forests versus Pre–Andean Amazon Forests and Yungas, respectively, shared the highest number of species, with index values around 0.5. The remaining comparisons showed intermediate similarity values ranging from 0.2 to 0.4.

### Roosts

We captured 29 bats representing 11 species from seven roosts across five study sites and four ecoregions (Table 3). The most diverse roost, Cueva Uarajo in Mamacona (Fig. 2), contained four species. The cave had an oblique entrance 1.5 m high, a single main chamber ~ 6 m high at its tallest point and 10 m wide, and a corridor extending to the right side that was 3 m wide and 5 m long. At the far right of the cave, near the entrance, a narrow passage ~ 0.5 m wide opened into a small chamber 2–3 m deep and 1 m high. Local residents reported that the cave had partially collapsed in recent months and was previously larger. During daytime surveys, we observed ~ 100 bats and focused capture efforts on the species detected inside the roost. The remaining roosts each contained one or two species, although the sampled *Ficus* tree was very large and structurally complex, suggesting that additional species may have been present. Roost sampling proved particularly important for documenting species detected only acoustically or not captured using other sampling methods (Table 3).

**Table 3.** Roosts surveyed, species recorded, and numbers of bats captured during the Identidad Madidi expedition. † = species captured exclusively in roosts, ‡ = species captured in roosts and also detected acoustically. Ecoregions: PAAF = Pre–Andean Amazon Forests, SAAF = Sub–Andean Amazon Forests, IADF = Inter–Andean Dry Forests, YU = Yungas.

**Roost**	**Species**	**# individuals captured**	**Locality, Ecoregion**
Abandoned house roof	*Molossus molossus*‡, *Myotis nigricans*	6, 1	Sipia, IADF
Cueva Uarajo	*Anoura peruana*, *Carollia perspicillata*, *Diphylla ecaudata*†, *Peropteryx macrotis*‡	8, 1, 2, 2	Mamacona, YU
Chapel	*Eumops auripendulus* †	2	Sipia, IADF
*Ficus* tree	*Carollia manu*, *Saccopteryx bilineata*‡	1, 2	Hondo Alto, SAAF
*Heliconia* leaf	*Thyroptera tricolor* †	1	Alto Madidi, PAAF
Robust tree trunk	*Myotis riparius*	1	Sipia, IADF
Slender tree trunk	*Myotis riparius*	2	Heath River, PAAF

### New country records

We report seven bat species recorded for the first time in Bolivia from Madidi and one that potentially represents an undescribed species. These records were documented through voucher specimens collected during fieldwork, acoustic detections with unequivocal vocal signatures, and one overlooked museum specimen.

#### Anoura aequatoris

BOLIVIA • 1 F; La Paz Department, Franz Tamayo Province, Apolo Municipality, Sipia, Suyo Suyo; 14.40286°S, 68.5485°W; 730 m a.s.l.; 1 Jul. 2015; L. Siles leg.; Río Machariapo; CBF 8681.

**Figure 6.**
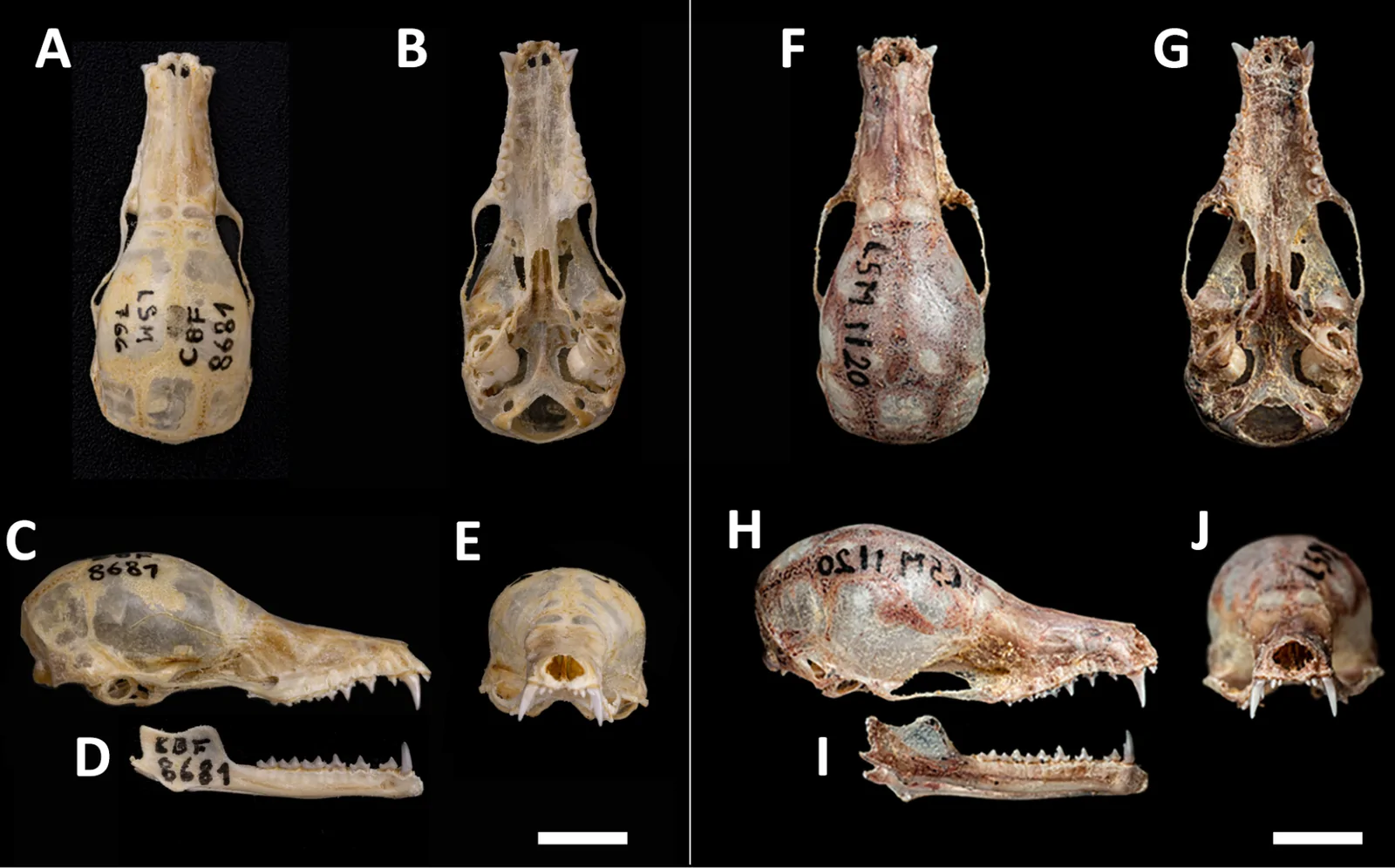
Cranial views of the new Bolivian records of *Anoura*. **A–E**. *A. aequatoris* (CBF 8681) from Sipia; **F–J**. *A. fistulata* (CBF 8684) from Sarayoj. **A, F**. Dorsal view of skull; **B, G**. Ventral view of skull; **C, H**. Lateral view of skull; **D, I**. Lateral view of mandible; **E, J**. Frontal view of skull. Photographs by Omar Torrico (WCS). Scale bar: 5 mm.

**Figure 7.**
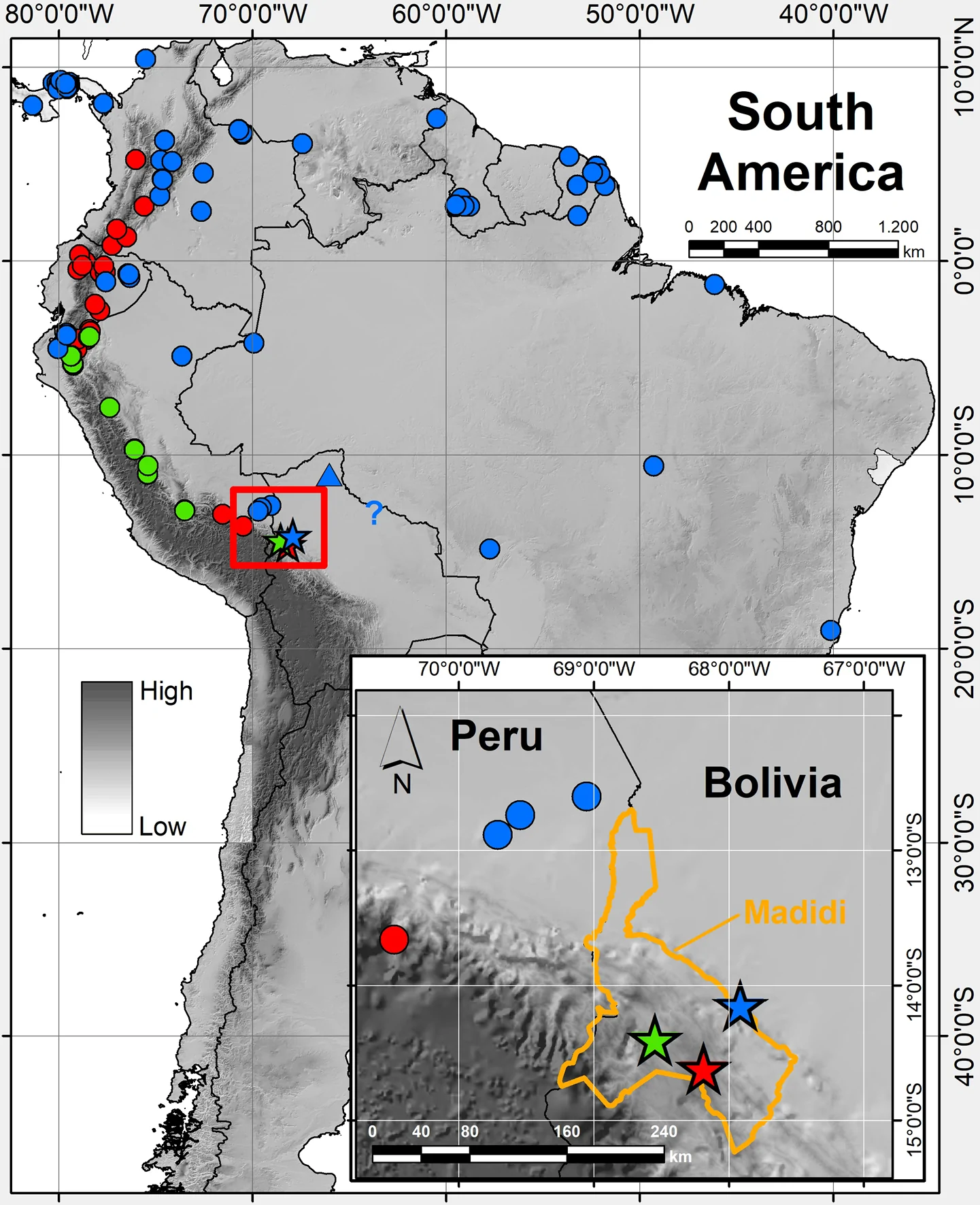
Collecting localities of new country records. *Anoura aequatoris* (green), *A. fistulata* (red), and *Molossus coibensis* (blue). Orange outline = Madidi. Stars = new records in Madidi, triangle = new record in Beni Department, ? = ambiguous locality on the Itenez River. Collecting localities based on the literature (circles): *A. aequatoris* (Pacheco et al. 2018), *A. fistulata* (Muchhala et al. 2005; Lee et al. 2008, 2010; Mantilla-Meluk and Baker 2008; Rex et al. 2008; Mantilla-Meluk et al. 2009; Gárate-Bernardo and Carrasco-Rueda 2011; Calderón-Acevedo and Muchhala 2018; Pacheco et al. 2018), and *M. coibensis* (Corrêa da Costa et al. 2013; GBIF.org 2026).

A previous record of the Ecuadorean Tailless Bat *Anoura aequatoris* (Phyllostomidae, Glossophaginae) from Bolivia (Mantilla-Meluk et al. 2012) was based on a single specimen (MSB 68350), whose identification was unconfirmed by Pacheco et al. (2018). Consequently, the occurrence of *A. aequatoris* in Bolivia remained uncertain. The specimen (Figs 6A–E, 7) was identified based on the following diagnostic characters: lower lip extends 2.5 mm beyond upper lip, uropatagium narrow (3.5 mm) and fringed with long hairs, dark brown posterior claws, distinct sulcus in canine, short rostrum length, and hypoconal basin of M1 not shallow and forming a less obtuse angle (Pacheco et al. 2018; Díaz et al. 2021). External measurements (mm) were total length 67, tail length 5, hind foot length 10, ear length 14, and forearm length 36.48. Craniodental measurements (mm) were greatest skull length 22.75, condylobasal length 21.65, palatal length 11.76, maxillary toothrow length 8.28, zygomatic width 9.18, braincase width 8.6, postorbital width 4.31, palatal width at M3 5.45, palatal width at canines 3.84, braincase height 6.47, dentary length 16.08, mandibular toothrow length 8.64, and dentary height 1.49. All measurements, except greatest skull length, fall within the range reported for *A. aequatoris* by Pacheco et al. (2018).

#### Anoura fistulata

BOLIVIA • 1 F (pregnant); La Paz Department, Franz Tamayo Province, Apolo Municipality, Sarayoj; 14.61738°S, 68.18829°W; 1162 m a.s.l.; 22 Sep. 2017; L. Siles leg.; Río Cargadero; CBF 8684.

The Tube-lipped Tailless Bat *Anoura fistulata* (Phyllostomidae, Glossophaginae) was previously reported for Bolivia by Aguirre et al. (2019) based on the specimen reported here, but was subsequently excluded from later checklists because the voucher had not yet been deposited in a scientific collection (Poma-Urey et al. 2023; Aguirre et al. 2026). The specimen’s (Figs 6F–J, 7) soft-tissue diagnostic characters, including lower lip and tongue length, were recorded in the field while the specimen was fresh. The lower lip extended 4 mm beyond the upper lip and the tongue measured 63 mm, both well within the range reported for *A. fistulata* (lower lip = 3.3–4.8 mm and tongue = 60–80 mm; Muchhala et al. 2005; Díaz et al. 2021). We additionally used the morphological characters described by Pacheco et al. (2018) to identify the specimen, including a wide interfemoral membrane (>4 mm; 6 mm in our specimen), shallow palatal shape, curved zygomatic arch in lateral view, distinct lambdoid crest, outer upper incisors longer than inner upper incisors, large mandibular symphysis, slightly curved mandible ventral margin, and large mental foramen (Fig. 6F–J). Morphometrically, the external and craniodental measurements of our specimen fall within the range reported for *A. fistulata* from Ecuador, Colombia, and Peru, with two exceptions: total length, slightly larger in our specimen, and condylobasal length, slightly smaller in our specimen (Table 4). We interpret these differences as intraspecific variation.

**Table 4.** External and cranial measurements of *Anoura fistulata* reported in Madidi. The female voucher (CBF 8684) is compared with measurement ranges reported in the literature. The ranges reported by Muchhala et al. (2005) and Calderón-Acevedo and Muchhala (2018) include both sexes, whereas Pacheco et al. (2018) reported ranges for males only. Bol = Bolivia, Ecu = Ecuador, Col = Colombia, Per = Peru. Measurements are in mm and sample sizes in parenthesis.

**Measurements**	**CBF 8684 (Bol)**	**Muchhala et al. 2005 (Ecu)**	**Calderón-Acevedo and Muchhala 2018 (Col, Ecu)**	**Pacheco et al. 2018 (Ecu, Per)**
Weight (g)	11.00	9.5–17.5 (12)		12.60–13.00 (2)
Total length	69.00	57.0–67.0 (13)		
Tail length	5.00	4.0–7.0 (10)		0.00–6.00 (3)
Foot length	11.00	7.0–11.5 (12)		9.00–13.00 (4)
Ear length	14.00	12.3–17.0 (14)		14.00–17.00 (4)
Forearm length	37.87	35.0–40.0 (19)	36.63–38.26 (3)	35.00–40.00 (10)
Greatest skull length	22.34	22.9–26.1 (6)	22.11–25.19 (4)	22.60–26.00 (11)
Condylobasal length	21.70	22.30–25.10 (6)	21.95–24.46 (4)	22.10–25.30 (11)
Zygomatic breadth	9.70	9.40–10.30 (3)		9.40–10.30 (7)
Mastoid breadth	9.57		9.54–9.94 (4)	
Braincase breadth	9.04	9.20–9.70 (6)	9.11–9.42 (4)	8.92–9.83 (11)
Postorbital breadth	4.52	4.40–5.1 (6)	4.47–5.02 (4)	4.43–4.95 (11)
Maxillary toothrow length	8.37	8.10–9.60 (6)	8.34–8.89 (4)	8.40–9.80 (11)
Breadth across molars	5.58	4.90–5.9 (6)	5.22–5.73 (4)	5.30–6.27 (11)
Mandible length	16.38	16.50–19.30 (6)	15.89–18.23 (4)	16.20–19.50 (11)
Mandibular toothrow length	8.72	8.40–9.80 (6)	8.84–9.57 (4)	8.53–10.34 (11)

#### *Histiotus* sp.

Several specimens of the genus *Histiotus* (Vespertilionidae) collected at two localities may represent an undescribed species. One female was collected at Chullo on 18 Nov. 2017 (14.68914°S, 69.04917°W; 2,863 m a.s.l.), and four males were collected at Chokollo on 23 and 24 Nov. 2017 (14.73689°S, 69.21736°W; 4,857 m a.s.l.). These specimens exhibit morphological characters that distinguish them from all currently recognized species of *Histiotus* and are currently under analysis.

#### Molossus coibensis

This free-tailed bat species (Molossidae) is rarely captured (Carrasco-Rueda et al. 2021), and no specimens are currently known in Bolivian scientific collections. However, the database of the Smithsonian Institution, National Museum of Natural History (USNM) contains 30 specimens identified as *Molossus coibensis* from Bolivia. Twenty-nine were collected in the Department of Beni, whereas one originated from the locality of Tumupasa, Department of La Paz, Iturralde Province (Fig. 7). The locality is listed in the database as “Trumupasa”, which appears to be a misspelling of Tumupasa. We georeferenced this record to 14.1500°S, 67.9167°W at 1,076 m a.s.l. The voucher (USNM 238690) is preserved as a skull, with the remainder of the specimen in fluid, and was collected by W. M. Mann on an unknown date. We were unable to examine the specimen directly and therefore relied on the current identification provided in the USNM database.

The USNM database lists *Molossus molossus cherrei* as a previous identification for this specimen. The current identification as *M. coibensis* was last modified on 8 Apr. 2020, coinciding with recent taxonomic revisions of the genus *Molossus* (e.g., Loureiro et al. 2018, 2020). Although the basis for the reidentification is not documented in the database, the timing suggests that the specimen was reassessed under the revised taxonomy. As currently identified, USNM 238690 represents a previously overlooked record of *M. coibensis* from Bolivia.

The following four species were documented exclusively through acoustic recordings. Although some of these taxa had been reported previously from Bolivia, they were excluded from recent revisions of the country’s bat fauna because the available records lacked verifiable voucher material or acoustic documentation (Poma-Urey et al. 2023; Aguirre et al. 2026). The recordings presented here include diagnostic call structure, quantitative parameters, and voucher files deposited in the open-access repository Zenodo (doi: 10.5281/zenodo.21300753), providing material for independent evaluation of the acoustic identifications.

#### Cormura brevirostris

**Figure 8.**
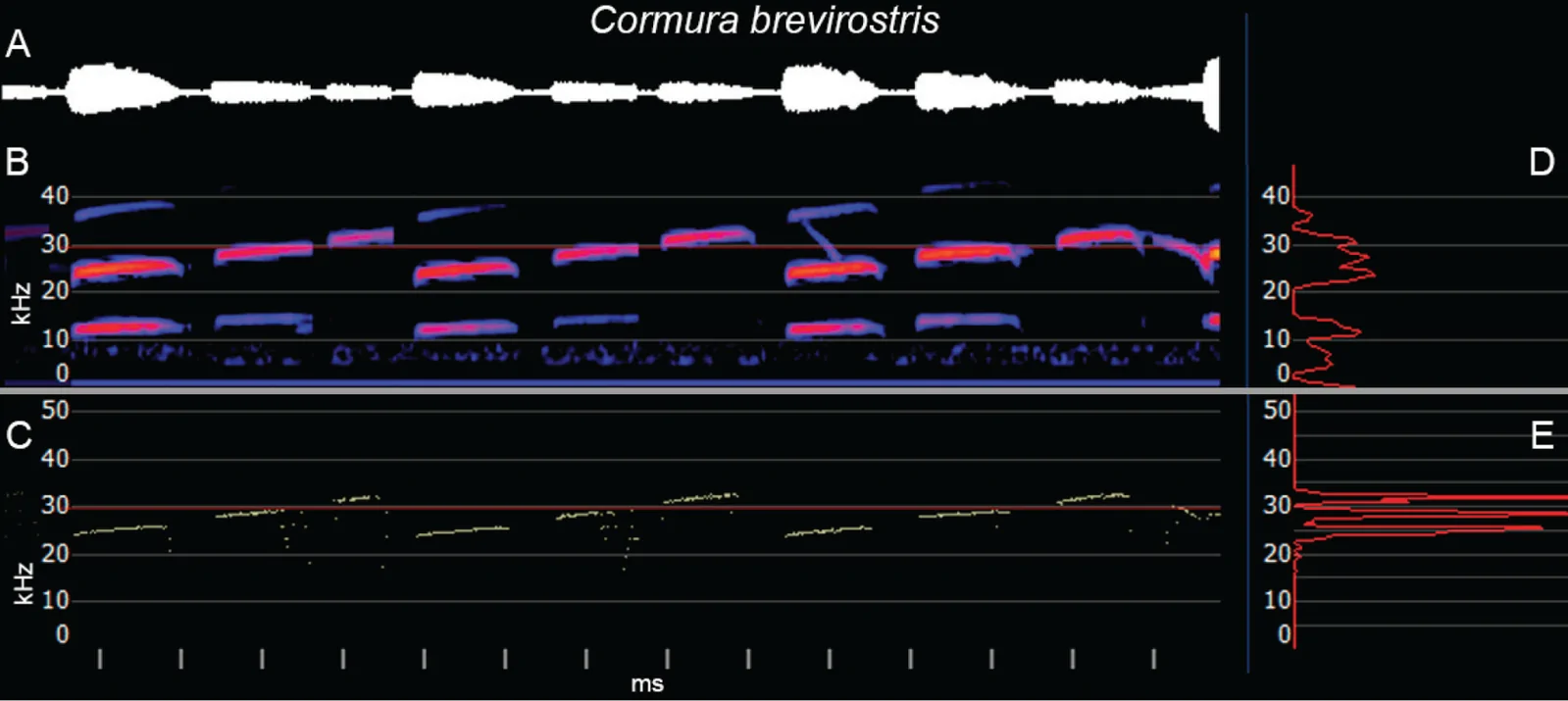
Representative echolocation calls of *Cormura brevirostris* recorded at Alto Madidi. **A**. Oscillogram showing a conical shape; **B**. Full-spectrum diagram showing all detected harmonics; **C**. Zero-crossing plot from which call measurements were obtained; **D**. Power graph showing the full-spectrum distribution of acoustic energy; **E**. Cycles graph showing the corresponding F_CG measurement derived from the zero-crossing signal. Interpulse intervals are not shown.

The Chestnut Sac-winged Bat *Cormura brevirostris* (Emballonuridae) was previously reported from Alto Madidi by Terán (2004) in an unpublished thesis and subsequently included in summaries of the Bolivian bat fauna (Vargas Espinoza 2007; Aguirre et al. 2019). However, the species was excluded from recent checklists because no verifiable voucher material or diagnostic acoustic documentation was available (Poma-Urey et al. 2023; Aguirre et al. 2026). We recorded this species once at Alto Madidi on 25 Oct. 2015 (13.63343°S, 68.74166°W; 249 m a.s.l.). The recording was identified based on the diagnostic characteristics described by Arias-Aguilar et al. (2018): FME in the second harmonic, an FM_u_/qCF_u_/FM_d_ call structure, regular alternation among three call types with values of FME_1_ ≈ 25–26, FME_2_ ≈ 28–29, and FME_3_ ≈ 31–32 kHz; and a characteristic conical shape in the oscillogram, with maximum amplitude occurring at the beginning of the pulse. The recording obtained in Madidi exhibited these characteristics (Fig. 8). Mean measurements from one recording were: call type I (n = 10 pulses), F_CG_1_ = 24.93 ± 0.34, Dur_1_ = 10.81 ± 1.71, Fmax_1_ = 25.26 ± 0.24, Fmin_1_ = 22.9 ± 0.50; call type II (n = 16), F_CG_2_ = 28.34 ± 0.68, Dur_2_ = 11.16 ± 1.19, Fmax_2_ = 28.79 ± 0.50, Fmin_2_ = 27.09 ± 0.48; and call type III (n = 9) F_CG_3_ = 32.14 ± 0.61, Dur_3_ = 11.77 ± 2.11, Fmax_3_ = 32.74 ± 0.46, Fmin_3_ = 30.29 ± 0.39. The acoustic characteristics and parameter values of this recording are consistent with published descriptions of *C. brevirostris* (Arias-Aguilar et al. 2018), supporting its identification and documenting the species acoustically in Bolivia.

#### Diclidurus isabella

**Figure 9.**
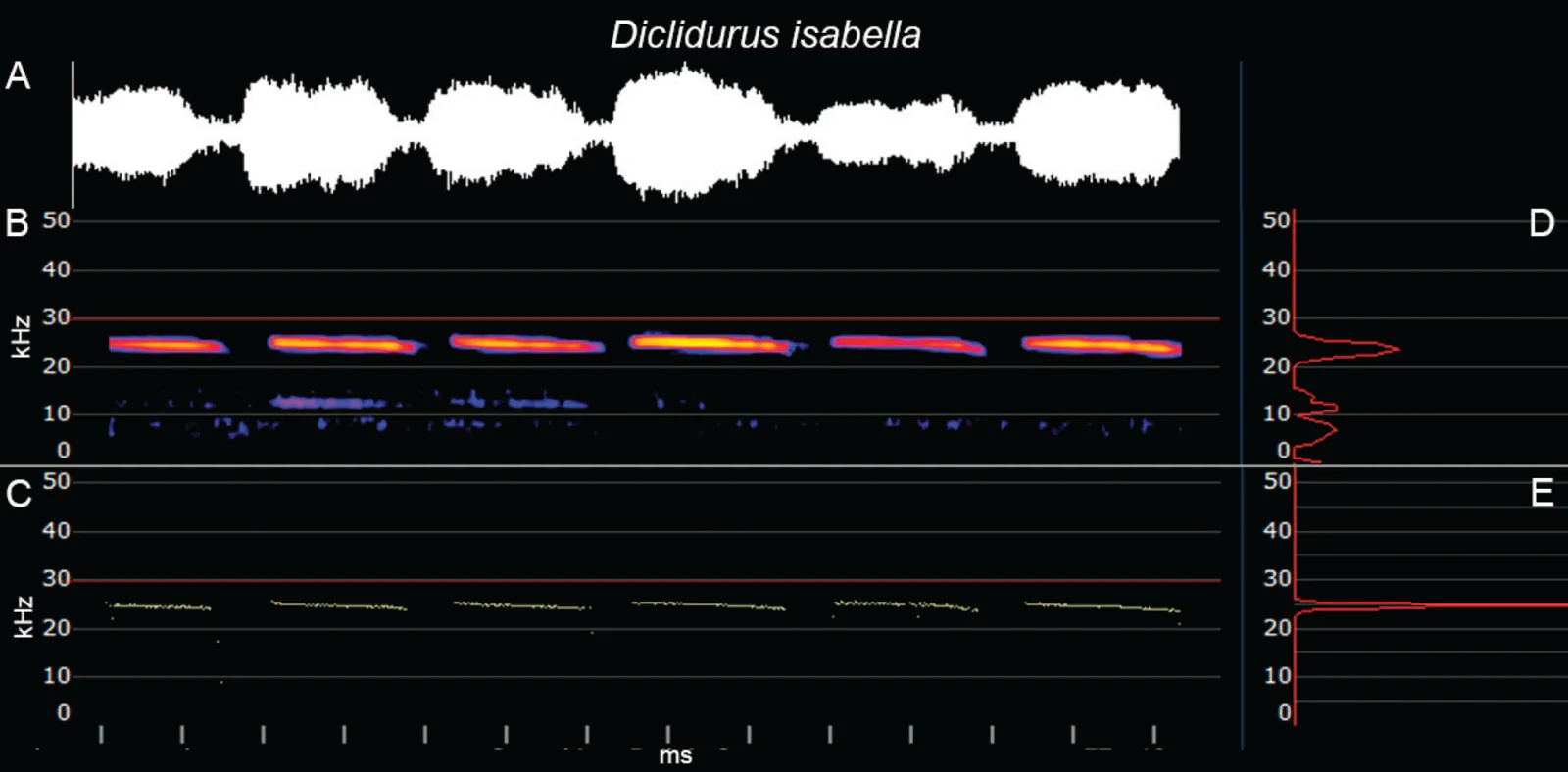
Representative echolocation calls of *Diclidurus isabella* recorded at Alto Madidi. **A**. Oscillogram showing little variation in each pulse; **B**. Full-spectrum diagram showing all detected harmonics; **C**. Zero-crossing diagram from which call measurements were obtained; **D**. Power graph showing the full-spectrum distribution of acoustic energy; **E**. Cycles graph showing the corresponding F_CG measurement derived from the zero-crossing signal. Interpulse intervals are not shown.

We recorded the Isabelline Ghost Bat *Diclidurus isabella* (Emballonuridae) at Alto Madidi on 13, 14, and 25 Oct. 2015 (13.633°S, 68.742°W; 246 m a.s.l.), at Heath Bosque on 24 Jun. 2017 (12.73274°S, 68.77873°W; 187 m a.s.l.), and at Heath Pampa on 8 Jul. 2017 (13.02098°S, 68.84155°W; 210 m a.s.l.). We deposited 15 WAV files from these three localities in Zenodo. Acoustic characteristics of the genus *Diclidurus* were evaluated following Arias-Aguilar et al. (2018), and species-level identification was based on the acoustic characterization provided by Miller et al. (2023). Characteristics of *Diclidurus* include FME in the second harmonic, regular alternation between two call types or no alternation, a qCF call structure with initial and final FM components that are barely perceptible or absent, irregular call duration, and FME always below 32 kHz. In the oscillogram, pulses show a maximum amplitude usually at the beginning of the call, and little variation throughout the pulse. The recordings obtained in Madidi exhibited these characteristics (Fig. 9). Miller et al. (2023) identified the characteristic frequency (Fc) as a diagnostic parameter for distinguishing *D. isabella* from *D. albus* (Table 5), a species with which it could be confused in the region. Mean acoustic parameters were as follows: call type I, based on 115 pulses from 15 recordings, F_CG_1_ = 24.83 ± 0.31, Dur_1_ = 18.82 ± 3.91, Fc_1_ = 24.34 ± 0.37, Sc_1_ = 3.08 ± 2.87, Fmax_1_ = 25.47 ± 0.37, Fmin_1_ = 23.51 ± 0.53; and call type II, based on nine pulses from six recordings, F_CG_2_ = 26.57 ± 0.47, Dur_2_ = 15.71 ± 5.55, Fc_2_ = 26.06 ± 0.63, Sc_2_ = 4.06 ± 1.88, Fmax_2_ = 27.43 ± 0.69, Fmin_2_ = 25.40 ± 0.75. The acoustic characteristics and parameter values of our recordings are consistent with those reported for *D. isabella* by Miller et al. (2023), supporting the identification and documenting the first record of the species in Bolivia.

**Table 5.** Comparison of the diagnostic characteristic frequency (Fc, kHz) in *Diclidurus albus* and *D. isabella* based on Miller et al. (2023), and in call type I recorded in Madidi (this study). Pulse sample size (N), mean ± standard deviation (SD), minimum – maximum range, and 10^th^ – 90^th^ percentiles are provided.

	***D. albus***	***D. isabella***	
	**Miller et al. 2023**	**Miller et al. 2023**	**This study**
N	24,626	261	115
Fc mean ± SD	21.6 ± 0.6	25.4 ± 1.1	24.34 ± 0.37
Fc min – max	20–23.5	24–28	23.32–25.16
Fc 10–90%	20.8–22.4	24.1–27.1	24.30–24.38

#### Molossus currentium

**Figure 10.**
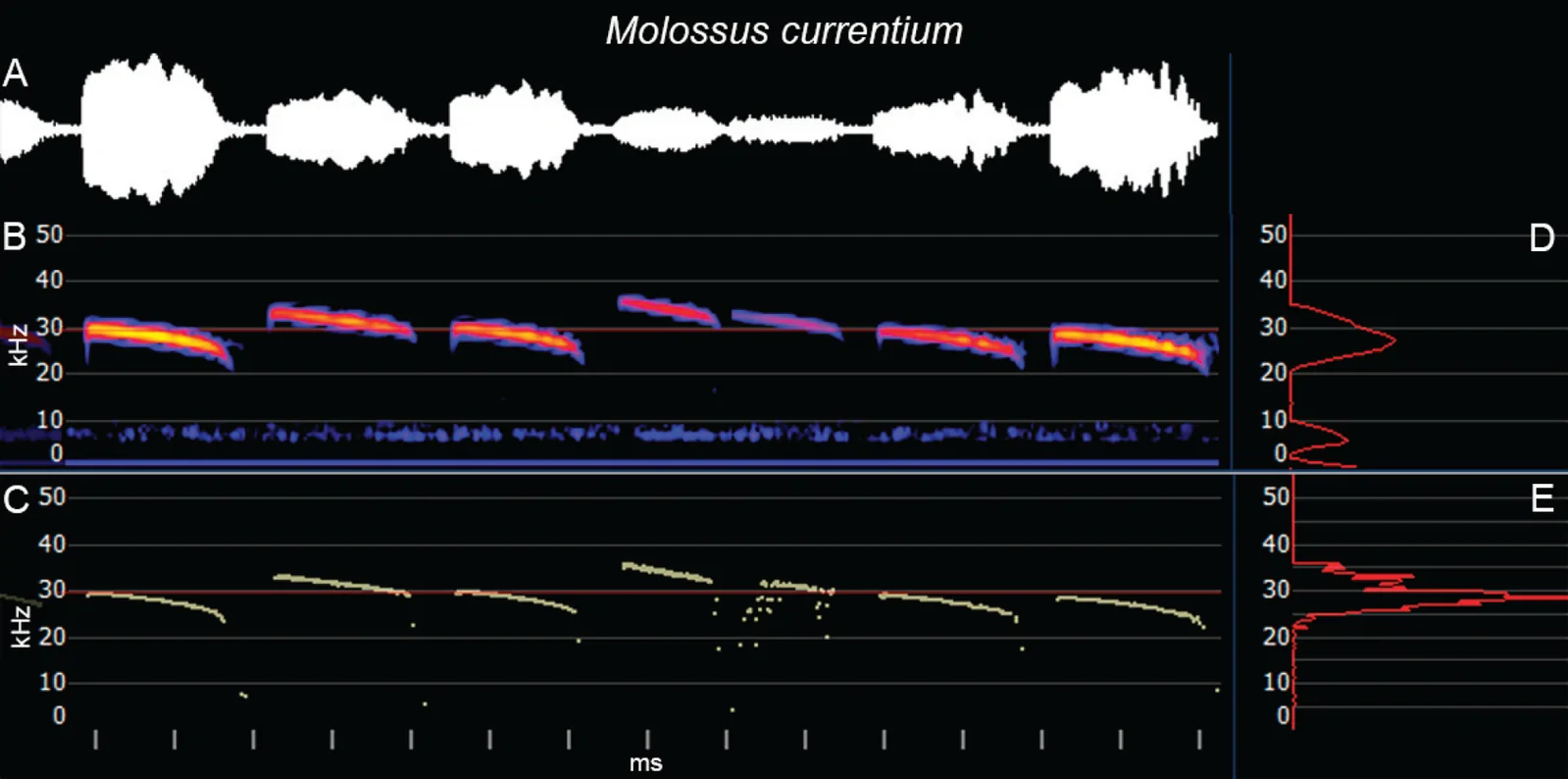
Representative echolocation calls of *Molossus currentium* recorded at Alto Madidi. **A**. Oscillogram; **B**. Full-spectrum diagram; **C**. Zero-crossing plot from which call measurements were obtained; **D**. Power graph showing the full-spectrum distribution of acoustic energy; **E**. Cycles graph showing the corresponding F_CG measurement derived from the zero-crossing signal. Interpulse intervals are not shown.

The Thomas’s Mastiff Bat *Molossus currentium* (Molossidae) was previously reported for Bolivia by Aguirre et al. (2019) based on acoustic recordings and voucher specimens that were subsequently lost (L. F. Aguirre, pers. comm., 8 May 2026). The species was excluded from recent checklists because no verifiable voucher specimens or diagnostic acoustic recordings were available (Poma-Urey et al. 2023; Aguirre et al. 2026). We recorded this species at four sites over an elevation range of 187–1,180 m a.s.l.: Alto Madidi on 14 and 25 Oct. 2015 (13.633°S, 68.741°W; 248 m a.s.l.), Heath Bosque on 24 Jun. 2017 (12.73274°S, 68.77873°W; 187 m a.s.l.), Heath Pampa on 8 Jul. 2017 (13.02098°S, 68.84155°W; 210 m a.s.l.), and Sarayoj on 16 Sep. 2017 (14.61606°S, 68.19025°W; 1,180 m a.s.l.). We deposited 40 WAV files from these four localities in Zenodo, five of these recordings had corresponding ZC files that were used for measurement of parameters. Species identification was based on the diagnostic acoustic characters described by Arias-Aguilar et al. (2018): FME in the fundamental harmonic; a call shape of (FM_u_)/qCF_d_, with a narrow bandwidth (<10 kHz); and frequency alternation of two call types, occasionally three, with values of FME_1_ ≈ 30, FME_2_ ≈ 33, and FME_3_ ≈ 36 kHz. The recordings obtained in Madidi exhibited these characteristics (Fig. 10). Mean acoustic parameters from five recordings were as follows: call type I, based on 83 pulses, F_CG_1_ = 29.44 ± 1.35, Dur_1_ = 16.87 ± 3.56, Fmax_1_ = 30.96 ± 1.61, Fmin_1_ = 26.0 ± 2.21; call type II, based on 46 pulses, F_CG_2_ = 32.94 ± 0.83, Dur_2_ = 16.50 ± 4.75, Fmax_2_ = 35.59 ± 2.38, Fmin_2_ = 30.53 ± 2.07; and call type III, based on 13 pulses, F_CG_3_ = 35.83 ± 1.39, Dur_3_ = 17.77 ± 7.47, Fmax_3_ = 40.93 ± 3.53, Fmin_3_ = 32.46 ± 1.32. The acoustic characteristics and parameter values of our recordings are consistent with published descriptions of *M. currentium* (Arias-Aguilar et al. 2018), supporting its identification and documenting the species acoustically in Bolivia.

#### Peropteryx kappleri

**Figure 11.**
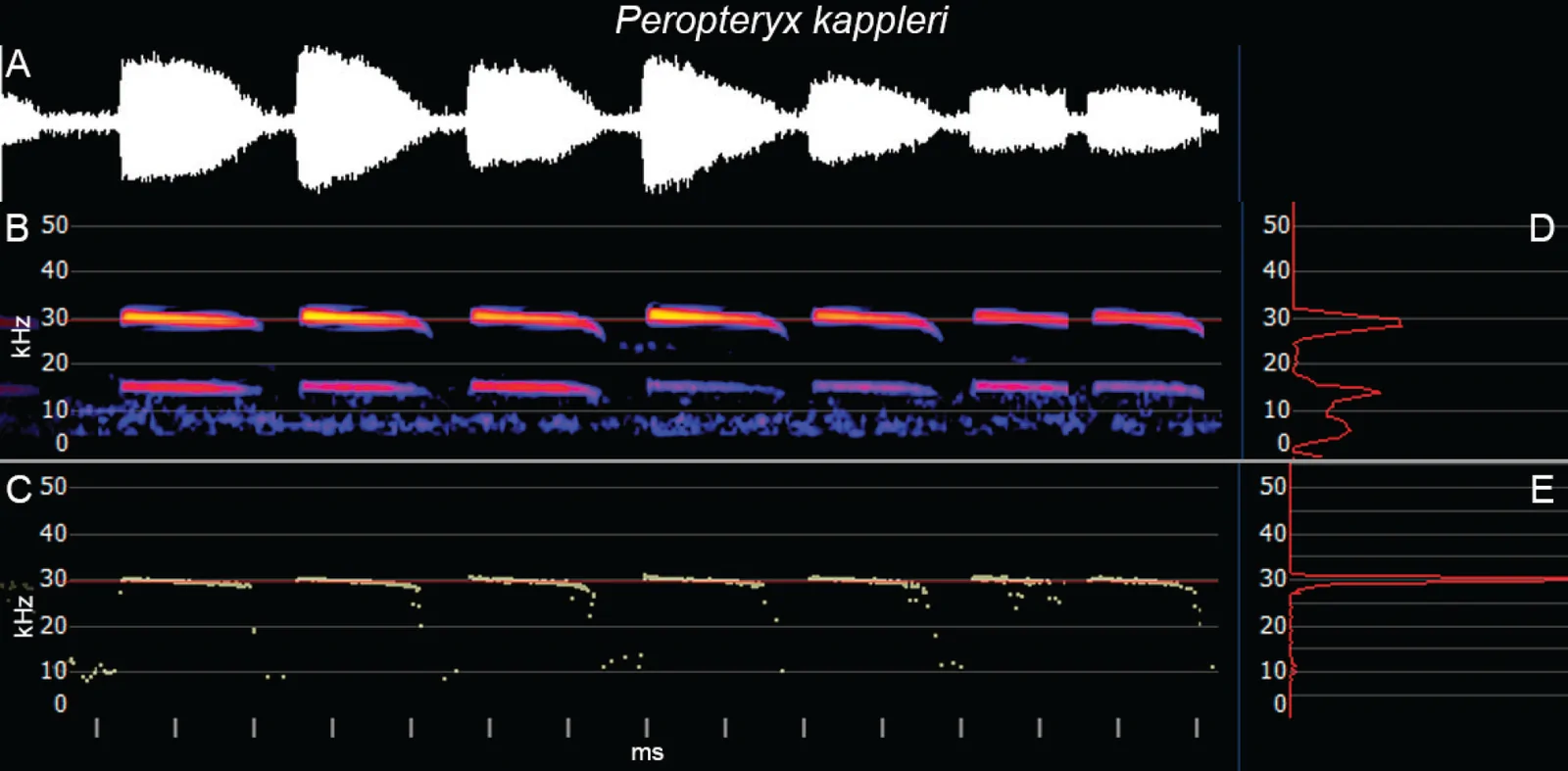
Representative echolocation calls of *Peropteryx kappleri* recorded at Alto Madidi. **A**. Oscillogram showing a triangular shape in each pulse; **B**. Full-spectrum diagram showing all detected harmonics; **C**. Zero-crossing plot from which call measurements were obtained; **D**. Power graph showing the full-spectrum distribution of acoustic energy; **E**. Cycles graph showing the corresponding F_CG measurement derived from the zero-crossing signal. Interpulse intervals are not shown.

Previous records of the Greater Dog-like Bat *Peropteryx kappleri* (Emballonuridae) in Bolivia were based on a specimen subsequently reidentified as *P. leucoptera* (Aguirre and de Urioste 1994; Poma-Urey et al. 2023) and acoustic records from Madidi (Terán 2004) and eastern Bolivia (Emmons et al. 2006). The species was excluded from recent checklists because no verifiable voucher specimens or diagnostic acoustic recordings were available (Poma-Urey et al. 2023; Aguirre et al. 2026). We recorded the species at Alto Madidi on 13 and 14 Oct. 2015 (13.633°S, 68.7415°W; 247 m a.s.l.); Hondo Alto on 30 Apr. 2016, and 5–6 May 2016 (14.634°S, 67.851°W; 320 m a.s.l.); Heath Bosque on 3 Jul. 2017 (13.02968°S, 68.85496°W; 215 m a.s.l.); and Heath Pampa on 10, 12, and 13 Jul. 2017 (13.02098°S, 68.84155°W; 210 m a.s.l.). We deposited 4 WAV files from these four localities in Zenodo. Species identification was based on the diagnostic acoustic characters described by Arias-Aguilar et al. (2018): FME in the second harmonic; absence of frequency alternation; qCF call component lacking upward or downward modulation; a well-defined terminal FM component; an absent or weakly developed initial FM component; a characteristic triangular pulse shape in the oscillogram, with maximum amplitude at the beginning of the pulse followed by a sharp decline; and FME values between 29 and 32. The recordings obtained in Madidi exhibited these characteristics (Fig. 11). Mean acoustic parameters from four recordings comprising 84 pulses were: F_CG = 29.83 ± 1.46, Dur = 10.75 ± 2.87, Fmax = 30.47 ± 1.00, and Fmin = 28.79 ± 1.61. The call characteristics and parameter values of our recordings are consistent with published descriptions of *P. kappleri* (Arias-Aguilar et al. 2018), supporting its identification and documenting the species acoustically in Bolivia.

## Discussion

The most recent account of Bolivian mammals recognizes 139 bat species (Aguirre et al. 2026). The seven new country records documented here increase that total to 146 recognized species. In addition, we report a potentially undescribed species of *Histiotus* that is currently under study. Madidi harbors 99 confirmed bat species, representing 68% of the bat fauna currently known from Bolivia and the highest richness documented for any protected area in the country. For comparison, Noel Kempff Mercado National Park has 60 confirmed species (Emmons et al. 2006), followed by Carrasco National Park (54; Vargas et al. 2005), Pilón Lajas Biosphere Reserve (35; Flores-Saldaña 2008), Kaa Iya National Park and Natural Integrated Management Area (31; Siles et al. 2004), and Cotapata National Park and Natural Integrated Management Area (29; Moya et al. 2008). Although these inventories may increase with additional sampling and historical records, Madidi currently exceeds all other protected areas by a substantial margin. Noel Kempff Mercado is the closest comparison, with richness predicted to exceed 90 species using the Chao1 estimator (Emmons et al. 2006), although that estimate included unidentified and acoustically detected taxa that would not meet the verification criteria adopted in recent national species compilations.

With 99 confirmed species, Madidi also ranks among the most bat-rich protected areas worldwide. Caura National Park in Venezuela has 108 confirmed species (Ochoa et al. 2009; Rivas and Ferrer 2012), followed by Yasuní Biosphere Reserve in Ecuador (94; K. Swing, pers. comm., 24 May 2026), Manu Biosphere Reserve in Peru (92 confirmed and up to 129 estimated; Patterson et al. 1996; Solari et al. 2006), Kanuku Mountains Protected Area in Guyana (89; Montambault and Missa 2002), and Iwokrama Forest in Guyana (88; Gilroy et al. 2015). At the local scale, Tiputini Biodiversity Station within Yasuní has an estimated 100 sympatric species (Rex et al. 2008; Bass et al. 2010), suggesting that total richness across the broader Yasuní landscape is likely considerably higher.

Species richness estimators indicate that additional species remain undetected in Madidi. ACE estimator predicted 95.67 species, Chao2 105, and Jackknife2 115.91. While ACE is often more independent of data aggregation, it can provide a conservative lower bound for richness (Hortal et al. 2006). The divergence between ACE and Jackknife2 values, together with the non-asymptotic rarefaction curve, indicates that the dataset contains a high proportion of rare species or singletons (Hortal et al. 2006). This suggests that Madidi’s habitat heterogeneity likely harbors additional cryptic or low-density species that require long-term sampling to detect. Furthermore, several ecoregions, particularly Cerrado Paceño, Inter-Andean Dry Forests, and High Andean Vegetation, were represented by few sampling sites, while seasonal variation in activity may also have affected detectability (Meyer et al. 2011). Future research should prioritize undersampled ecoregions and conduct multi-season surveys to capture shifts in species composition.

The exceptional richness of Madidi appears to result largely from species turnover across its extensive elevational gradient and diverse ecoregions (beta diversity). The strongest pattern involved High Andean Vegetation, probably reflecting environmental filtering associated with low temperatures, reduced primary productivity, and limited food and roost resources. However, this result should be interpreted cautiously because High Andean Vegetation was represented by a single locality, where only two species were documented and acoustic sampling was unavailable. Its distinctiveness may therefore partly reflect sampling effort and detectability artifacts (Meyer et al. 2011, 2014). Among more thoroughly sampled ecoregions, Inter–Andean Dry Forests and Yungas shared less than one-third of their species despite being geographically adjacent, further indicating that environmental conditions and habitat structure strongly influence bat distributions within Madidi. Similar patterns of turnover associated with environmental heterogeneity and elevation have been reported at broader geographic scales despite the high vagility of bats (López-González et al. 2015).

The high diversity documented in Madidi also reflects the combination of data sources and survey methods. For the baseline, the compilation of information from technical reports, theses, and museum specimens proved particularly valuable in a country where much biodiversity information exists but remains unpublished (Amano and Sutherland 2013; De la Cruz Prego 2021). Fieldwork using mist-netting, harp-trapping, roost searches, and acoustic surveys documented 83 species, whereas combining these records with historical data and museum specimens increased the total inventory to 100 taxa. Three species found in roosts were not detected by other methods, although systematic searches for modified leaf tents were not conducted because roosts searches were opportunistic. Acoustic sampling added 14 species, highlighting its importance for non-phyllostomids and aerial insectivores that often forage above mist-net height and can detect and avoid nets (Kalko and Handley 2001; Arias-Aguilar et al. 2018). The value of combining complementary methods is consistent with other bat inventories that emphasize acoustic sampling as an important component of community surveys (Appel et al. 2021; Barbosa Leal et al. 2022; Mancini et al. 2024).

The increasing use of acoustic methods also underscores the importance of archiving voucher recordings in publicly accessible repositories. Three new acoustic records reported here had previously been mentioned for Bolivia but were excluded from recent taxonomic revisions because the original recordings and diagnostic information were unavailable for verification (Poma-Urey et al. 2023; Aguirre et al. 2026). *Diclidurus* provides a clear example. The publication of diagnostic acoustic parameters for all species in the genus (Miller et al. 2023) allowed us to confirm *D. isabella* in Madidi (Table 5), whereas the absence of historical voucher recordings prevents the reassessment of most previous Bolivian records. Consequently, the identity of records previously assigned to *D. albus* (e.g., Emmons et al. 2006) remains unresolved.

Museum specimens provide similarly important permanent evidence that can be reexamined as taxonomy changes. The two *Anoura* species reported here illustrate this point well. The only previously reported Bolivian specimen of *A. aequatoris* (Mantilla-Meluk et al. 2012) was considered unconfirmed by Pacheco et al. (2018). Subsequently, *A. aequatoris* was treated as a synonym of *A. caudifer* (Calderón-Acevedo et al. 2022), a treatment that was adopted in a Bolivian bat checklist (Poma-Urey et al. 2023), and later recognized again as a distinct species (Molinari et al. 2023; Simmons and Cirranello 2026). Our record extends its distribution 562.56 km SE of the nearest locality at Campamento Wayrapata, Cusco, Peru (locality 6 in Pacheco et al. 2018). Similarly, the specimen on which the Bolivian record of *A. fistulata* was based (Mantilla-Meluk et al. 2014) was later reidentified as *A. caudifer* (Calderón-Acevedo and Muchhala 2018). Our record extends the distribution of *A. fistulata* 269 km SE of the nearest locality in Peru (Gárate-Bernardo and Carrasco-Rueda 2011). Both records represent the southernmost known occurrences (Fig. 7). Together, they bring the number of *Anoura* species recorded in Bolivia to seven: *A. aequatoris*, *A. caudifer*, *A. cultrata*, *A. fistulata*, *A. geoffroyi*, *A. latidens*, and *A. peruana* (this study; Aguirre et al. 2026), five of which occur in Madidi.

Museum collections can also reveal overlooked records as taxonomic knowledge improves. Our record of *Molossus coibensis* is based on a USNM specimen that, together with 29 others from Beni Department, appears to have been reidentified as *M. coibensis* in 2020 following recent taxonomic revisions of the genus (Loureiro et al. 2018, 2020). Although we could not examine the Tumupasa specimen directly, its occurrence in Bolivia is consistent with the known distribution of the species and nearby records from Peru (Carrasco-Rueda et al. 2021) and Brazil (Corrêa da Costa et al. 2013). These records fill a previously undocumented gap in the distribution of *M. coibensis* in southwestern Amazonia (Fig. 7), suggesting that its occurrence in Bolivia was biogeographically expected but previously overlooked. Collectively, the new records reported here demonstrate that additions to the Bolivian bat fauna can still result from the examination of museum specimens, acoustic sampling, and advances in taxonomic knowledge.

*Gardnerycteris koepckeae* also warrants mention because the specimen reported by Siles and Wallace (2021), collected during the Identidad Madidi expedition at 2,280 m a.s.l. in Cargadero, Yungas ecoregion (CBF 8683), represents the highest elevational and southernmost record currently known for the species. Previously considered endemic to Peru and known from only three localities (Gardner and Patton 1972), *G. koepckeae* remained unrecorded for four decades before additional specimens were documented in Peru (Hurtado and Pacheco 2014; Carrasco-Rueda et al. 2022), Colombia (Morales-Martínez et al. 2020), and Bolivia (Siles and Wallace 2021).

## Conclusions

Our results confirm Madidi as one of the most important areas for bat conservation and research in the Neotropics. The combination of extensive fieldwork, acoustic sampling, museum collections, and historical records revealed an exceptionally diverse bat fauna and improved our knowledge of species distributions and habitat use in the region, while also providing supplementary natural history observations on reproduction. The high species turnover observed among ecoregions highlights compositional differences across Madidi’s elevational gradient, with different landscape units contributing distinct components to the park’s overall bat richness. However, differences in sampling effort among ecoregions warrant caution when interpreting these patterns. These findings emphasize the importance of protecting the full elevational gradient of Madidi. In addition to harboring 68% of the bat fauna currently known from Bolivia, Madidi supports three Vulnerable species, three Near Threatened species, and 14 Data Deficient species classified at the national level (Acosta et al. 2026). The global significance of this fauna was recognized in 2022 through the designation of Madidi as an Area of Importance for Bat Conservation (AICOM) by the Latin American and Caribbean Bat Conservation Network (RELCOM; Siles and Wallace 2022). This designation was supported by data compiled and generated through the Identidad Madidi initiative. Although 100 bat taxa, comprising 99 recognized species and one potentially undescribed species, are now confirmed for Madidi, richness estimators and the rarefaction analyses indicate that additional sampling may yield further species. Continued surveys, particularly in underrepresented ecoregions and using complementary sampling methods, along with voucher and acoustic documentation and ongoing taxonomic research, will refine our understanding of this fauna. The dataset presented here provides a robust baseline for future ecological, taxonomic, and conservation studies in one of the world’s most biodiverse protected areas.

## Additional information

### Artificial Intelligence (AI) use

The authors accept full responsibility for the content of the manuscript, including the disclosure of any use of AI.

Regarding the use of AI in the preparation of this manuscript, the authors declare the following: GPT.

**Used for**: Language, style and writing.

A free version of ChatGPT (OpenAI) was used to improve grammar and language flow.

### Author ORCIDs

L. Siles https://orcid.org/0000-0001-8721-4033

M. Hidalgo-Cossio https://orcid.org/0000-0003-3898-8766

N. Bernal-Hoverud https://orcid.org/0000-0002-5688-1201

M. Ocampo https://orcid.org/0000-0001-7365-3350

R.B. Wallace https://orcid.org/0000-0001-7411-6338

### Data availability

All of the data that support the findings of this study are available in the main text or Supplementary Information.

## Supplementary materials

Supplementary material 1 Supplementary information 1 Data type xlsx https://binary.pensoft.net/file/1829674This dataset is made available under the Open Database License (http://opendatacommons.org/licenses/odbl/1.0/). The Open Database License (ODbL) is a license agreement intended to allow users to freely share, modify, and use this Dataset while maintaining this same freedom for others, provided that the original source and author(s) are credited. Lizette Siles, Marisol Hidalgo-Cossio, Nuria Bernal-Hoverud, Mauricio Ocampo, Robert B. Wallace

Supplementary material 2 Supplementary information 2 Data type xlsx https://binary.pensoft.net/file/1829675This dataset is made available under the Open Database License (http://opendatacommons.org/licenses/odbl/1.0/). The Open Database License (ODbL) is a license agreement intended to allow users to freely share, modify, and use this Dataset while maintaining this same freedom for others, provided that the original source and author(s) are credited. Lizette Siles, Marisol Hidalgo-Cossio, Nuria Bernal-Hoverud, Mauricio Ocampo, Robert B. Wallace

Supplementary material 3 Supplementary information 3 Data type xlsx https://binary.pensoft.net/file/1829676This dataset is made available under the Open Database License (http://opendatacommons.org/licenses/odbl/1.0/). The Open Database License (ODbL) is a license agreement intended to allow users to freely share, modify, and use this Dataset while maintaining this same freedom for others, provided that the original source and author(s) are credited. Lizette Siles, Marisol Hidalgo-Cossio, Nuria Bernal-Hoverud, Mauricio Ocampo, Robert B. Wallace

Supplementary material 4 Supplementary information 4 Data type docx https://binary.pensoft.net/file/1829677This dataset is made available under the Open Database License (http://opendatacommons.org/licenses/odbl/1.0/). The Open Database License (ODbL) is a license agreement intended to allow users to freely share, modify, and use this Dataset while maintaining this same freedom for others, provided that the original source and author(s) are credited. Lizette Siles, Marisol Hidalgo-Cossio, Nuria Bernal-Hoverud, Mauricio Ocampo, Robert B. Wallace
