# Supplementary material for: Bats (Chiroptera) from Madidi National Park: an integrative assessment from field surveys, museum collections, and historical records
Source: Zookeys. 2026 Nov 5;1296:231–62. doi: 10.3897/zookeys.1296.206387 (PMC13760342; doi:10.3897/zookeys.1296.206387)
Supplement: Supplementary file 1 [file zookeys-1296-231_article-206387__-s004.docx]

**Bats (Chiroptera) from Madidi National Park: an integrative assessment from field surveys, museum collections, and historical records**

**Supplementary Information S4**

Values of the dissimilarity index between ecoregions in Madidi based on fieldwork data. Ecoregions: CP = Cerrado Paceño, HAV = High Andean Vegetation, IADF = Inter-Andean Dry Forests, PAAF = Pre-Andean Amazon Forests, SAAF = Sub-Andean Amazon Forests, YU = Yungas.

|  | SAAF | PAAF | HAV | CP | YU |
| --- | --- | --- | --- | --- | --- |
| PAAF | 0.35 |  |  |  |  |
| HAV | 0.96 | 0.97 |  |  |  |
| CP | 0.57 | 0.53 | 1.00 |  |  |
| YU | 0.38 | 0.50 | 0.92 | 0.66 |  |
| IADF | 0.52 | 0.55 | 1.00 | 0.61 | 0.58 |
